# Iconicity Gives Communicators a Head-Start, Even if Only the Producer Experiences It

**DOI:** 10.1162/OPMI.a.327

**Published:** 2026-02-01

**Authors:** Robert Snider, Petros Kaklamanis, Gareth Roberts

**Affiliations:** Department of Linguistics, University of Pennsylvania, Philadelphia, PA, USA; Department of Linguistics, University of Cambridge, Cambridge, UK

**Keywords:** iconicity, communication, language, referential communication game, experimental semiotics, laboratory languages, audience design

## Abstract

Iconicity has increasingly come to be recognized as widespread in language. It plays a particularly important role in bootstrapping new referring expressions in existing languages and in the emergence of new languages and other communication systems. The basis of this role has long been assumed to depend primarily on transparency of the iconic signal for the receiver, who benefits from being better able to identify its meaning. But might there also be producer-side advantages, distinct from this transparency-based comprehension benefit, that support communication? We investigated this using an experimental referential communication game in which dyads used a novel signaling medium to communicate fruits and vegetables. We manipulated both whether the producer could generate iconic signals and whether the receiver saw iconic or arbitrary signals. Results suggested that there was an iconicity benefit via stability in production even if the receiver was unable to perceive the iconicity (and therefore unable to benefit from transparency). However, while this gave dyads a substantial head-start, the lack of a benefit from iconicity for the receiver meant dyads in this condition still performed significantly less well overall than dyads with full iconicity.

## INTRODUCTION

The term *iconicity* refers to the phenomenon where communicative forms resemble, or are motivated by, what they refer to; onomatopoeia (e.g. “oink”, “buzz”) is a familiar example of iconicity in vocal language (Dingemanse et al., 2020). There is evidence from both natural language (Imai & Kita, 2014; Sandler et al., 2011) and laboratory experiments involving non-linguistic communication systems (Roberts et al., 2015; Verhoef et al., 2016) that iconicity plays an important role not only in the emergence of new languages and communication systems, but also the grounding of new referring expressions in established languages, both signed (Baus et al., 2013) and vocal (Perlman et al., 2015). An obvious reason for this is that iconicity aids comprehension by making it easier for comprehenders to guess the meaning (a property referred to as *semantic transparency*; Auch et al., 2020). For example, it ought to be easier for someone unfamiliar with English bird names to guess which bird “cuckoo” refers to than “sparrow.” This **comprehension benefit** for novel forms aids the development—or *bootstrapping*—of new shared communication systems (Lockwood et al., 2016), a claim supported by evidence from experimental communication tasks and learning tasks (e.g., Perlman et al., 2015; Sato et al., 2020). The comprehension benefit of iconicity aids not just the receiver of a signal, but also the sender, because both benefit from successful communication. But does iconicity also provide a **production benefit** for the sender that is distinct from the comprehension benefit for the receiver? That is, does iconicity facilitate the generation of novel forms before they even reach the receiver?

While production benefits have been claimed (or at least implied) to exist alongside comprehension benefits in past literature—Perlman et al. (2015), for instance, suggested that iconicity facilitates both the creation and comprehension of novel forms—there is, to our knowledge, no experimental work directly separating the potential communicative benefit of iconicity for the sender in generating novel forms from its benefit to the receiver in interpreting novel forms.

In this paper, we present an experimental investigation into this question using a *laboratory language game* paradigm (Galantucci et al., 2012; Nölle & Galantucci, 2022; Roberts, 2017) in which we manipulated whether iconicity was available to only the sender of a signal, both the sender and the receiver, or neither. This allowed us to identify whether iconicity provides a communicative production benefit distinct from any comprehension benefit to the receiver. The experiment will be described below. In the remainder of this section we present further background on the phenomenon of iconicity and approaches to studying it, including laboratory language games.

### Iconicity

Over the last couple of decades it has been increasingly argued that iconicity plays a significant role not only in sign languages, where its role has long been acknowledged (e.g., Mandel, 1977), but also in vocal languages. This includes not only onomatopoeia (Laing, 2019) and ideophones (Dingemanse, 2012), but also more abstract forms of iconicity. For instance, reduplication—involving total or partial repetition of the phonological material of a morpheme (as in *pancake-shmancake* or *very very good*)—is productive in all language families and is very frequently iconic, with repetition of the form in such cases typically corresponding in the meaning space to some kind of increase, such as plurality, iteration, or intensity (Downing & Stiebels, 2012).

Iconicity in natural language is particularly common in communicative situations that necessitate bootstrapping. Muysken (2013) claimed, for instance, that iconicity is part of a set of language-independent universal principles that govern “improvised language behavior, such as radical foreigner talk or incipient pidgin construction” (p. 716). Furthermore, iconicity features prominently in *homesigns*, signed communication systems innovated by children who are deaf from birth and not exposed to sign language at home or within their communities (Cartmill et al., 2017; Coppola & Brentari, 2014). In such circumstances iconicity provides an obvious comprehension benefit, making it easier for people to infer what the sign refers to. It is thus not surprising that it has been widely observed in the emergence of new sign languages (e.g., Sandler et al., 2011). While there are no known analogous cases of natural vocal languages emerging de novo in recent history, several researchers have argued that iconicity likely played an important role in the origins of human vocal communication systems as well (e.g., Perlman et al., 2015). Over time, as languages and other communication systems become increasingly established and conventionalized, iconicity becomes less evident, and it has been argued—with experimental support—that this fading of iconicity may aid the emergence of combinatorial phonology, with which iconicity seems to be in tension (Roberts & Galantucci, 2012; Roberts et al., 2015; Verhoef et al., 2016). This tension derives from the fact that increased combinatoriality (by which is meant the organization of linguistic form in terms of a relatively small finite set of recombinable units) reduces the degrees of freedom available for iconically representing the world. However, this does not preclude the retention of iconicity in some areas of language, and other work has emphasized its continued ubiquity even in vocal languages, albeit often in more abstract forms (Dingemanse, 2012; Perlman, 2017).

#### Conceptions of Iconicity.

As iconicity has increasingly become a focus of research, it has been conceived of in a number of different ways. Dingemanse et al. (2020) noted, for instance, that researchers vary in whether they treat it as a property that can be either present or absent, a semiotic relationship that comes in kinds, or a scalar substance that varies in degree. Akita and Imai (2022) made a further distinction between *primary* and *emergent* iconicity. Primary iconicity concerns iconic relations that hold cross-linguistically (such as most onomatopoeia in vocal languages), while emergent iconicity concerns iconic relations that are specific to particular language systems, such as the association in Korean of /a/ with lightness and /i/ with darkness. By definition, primary iconicity should be expected to dominate in the emergence of a novel system (such as a new sign language or a laboratory-based communication system) where establishing signs that will be understood by one’s audience is an obvious challenge and the comprehension benefits of iconicity are in obvious demand.

Although examples of primary iconicity such as onomatopoeia tend to be more obviously identifiable as iconic, both kinds of iconicity have tended to be overlooked as features of modern vocal languages (Perniss et al., 2010). There are several likely reasons for this. First, as discussed in the prior section, iconicity is often less obvious in long-established languages because conventionalization makes it less apparent (Ahlner & Zlatev, 2010). Second, the traditional emphasis in the field has been on the arbitrariness of the linguistic sign (de Saussure, 1916/2013; Hockett, 1960). This trend is driven partly by a longstanding tendency within linguistics to focus on vocal languages to the exclusion of sign languages (Fischer, 2000), with the manual–visual modality long assumed to afford more opportunity for iconicity (Armstrong et al., 1995; Downing & Stiebels, 2012; Fay et al., 2014; Tomasello, 2010). However, this assumption has been challenged somewhat in recent work (Perlman & Cain, 2014; Perlman et al., 2015, 2018).

#### Iconicity and Cognition.

There have been several studies focusing on the impact of iconicity on language processing and acquisition. On the topic of language processing, Sidhu et al. (2020) found lexical decision benefits for iconic forms, including for what they called “non-onomatopoeic iconic words.” Furthermore, Vinson et al. (2015) argued that iconicity facilitates both recognition and production, as demonstrated in picture matching and picture naming experiments, respectively, conducted in British Sign Language. In contrast, the interplay of iconicity and memory is less straightforward. Sidhu et al. (2023) reported that iconic forms are remembered more easily, but are also more likely to be “false alarms”—participants “remembering” never-before-seen iconic forms. A possible explanation lies in the fact that iconic forms were rated by participants as feeling more familiar than arbitrary forms (Sidhu et al., 2023).

Similar cognitive advantages are observed when examining language acquisition. For instance, work with Japanese-speaking two- and three-year-old children found that the iconicity of Japanese mimetic forms aided generalization of verbs of motion to new actors (Imai et al., 2008); similar results were even found for English-speaking children exposed to the same forms (Kantartzis et al., 2011). Additionally, Sato et al. (2020) tasked adult participants with learning a miniature artificial sign language and found that participants learned form-meaning mappings more successfully when iconicity was present, such as when a sign meaning *eat* involved raising a hand to one’s mouth as opposed shaking one’s fists. However, no benefit from iconicity was observed when participants were tasked with reproducing the forms.

Benefits of iconicity in terms of language processing and acquisition are relevant to both comprehension and production. Reduced processing costs and increased semantic transparency—discussed earlier—are obviously relevant to the former. Nevertheless, these advantages also seem compatible with the latter. Indeed, there is more than one potential source of production benefits. One concerns *generating* or *coining* novel forms. If iconic forms come more readily to mind—i.e., are more likely to be activated—than arbitrary forms in the process of coining a new referring expression, then this would be a source of production benefit. Another benefit concerns *memory*; iconic forms may be easier to recall than non-iconic forms, which would make it easier for the producer to produce a similar form again later. Both of these potential benefits would contribute to greater *stability* over repeated productions. Forms that are easier to remember are more likely to be reused on future occasion; if they are forgotten, then a coining benefit should make it more likely that a similar iconic form will be generated on multiple occasions. Another way of looking at this is that iconicity provides an *anchor* in production. Provided the producer still has an iconic connection to the referent in mind when producing a signal, this should constrain variability in the form of the signal. As an example: There is no strong reason for an arbitrary manual gesture to involve a particular number of fingers; as a consequence, if there is no established convention, this might be expected to vary freely. As an iconic signal for the number two, on the other hand, the number of fingers is much more constrained, and we should expect it to be highly consistent across multiple instances of conveying “two”.

### Laboratory Language Games

Much work on iconicity, like most work on language in general, involves stimuli drawn from natural languages. While this approach has many advantages, not least the maintenance of ecological validity, it also comes with several disadvantages. The reliance on already existing stimuli means that ideal levels of experimental control are often impossible; other issues of control with natural-language stimuli arise as a result of participants’ variable (and often unpredictable and hard-to-measure) prior experience with the stimuli. A solution—and one that is particularly applicable to studies that require exposure to novel stimuli or the generation of novel forms—is to employ an experimental *laboratory language game* paradigm. This approach, often referred to as *Experimental Semiotics* (Galantucci, 2009; Nölle & Galantucci, 2022), typically involves having participants play games of different kinds (for typologies see Galantucci et al., 2012; Roberts, 2017) in which they are prevented from using a pre-existing natural language or other familiar communication system. Instead, they must either learn and use an artificial language created for the experiment (e.g., Li & Roberts, 2023; Raviv & Arnon, 2018) or they must construct a communication system in a novel medium (e.g., Fay et al., 2018; Roberts & Clark, 2020; Stevens & Roberts, 2019). An important factor that typically sets this approach apart from traditional artificial-language-learning approaches is the inclusion of a social component; either dyads or small groups of participants interact directly with each other (e.g., Raviv et al., 2019; Sneller & Roberts, 2018; Wade & Roberts, 2020) or, in *iterated learning* designs, participants learn the communication system through exposure to the communicative signals of previous “generations” of participants (e.g., Kirby et al., 2015; Roberts & Fedzechkina, 2018; Verhoef et al., 2014). Such designs are also used to investigate the transmission of cultural behaviors other than communication systems (e.g., Derex et al., 2015; Matthews et al., 2012).

Several laboratory language game experiments have focused on iconicity in particular, a common result being that iconicity helps participants to converge on shared signs, allowing for the bootstrapping of communication systems across a variety of modalities (e.g., Fay et al., 2014; Perlman et al., 2015; Tamariz et al., 2018). In the graphical domain, experiments using Pictionary-like tasks have found both in pairs (Garrod et al., 2007) and in larger communicative groups (Fay et al., 2010) that iconic forms are employed initially to ground communication before giving way to simpler, more arbitrary forms. In the auditory domain, Perlman et al. (2015) had pairs of participant play “vocal charades,” in which they conveyed meanings with non-linguistic vocalizations. Pairs quickly converged on shared communication systems, whose signals were later consistently matched with the correct meaning by naive participants, suggesting high levels of iconicity. Verhoef et al. (2015) found similar results in an iterated laboratory language task involving slide whistles.

Laboratory language games have also been used to compare the role of iconicity in communication to its role in learning and repetition. Tamariz et al. (2018) gave participants a miniature artificial language and tasked them with either reproducing words individually or using the words to communicate in a pair. Naive participants rated the outputs of the communication condition as a better fit for their intended meanings (i.e., as more iconically suitable) than those of the individual condition, suggesting that iconicity might be particularly important in communicative scenarios. Similarly, Kempe et al. (2021) found in another iterated-learning experiment that iconicity emerged particularly when opportunities for rote memorization were minimal.

There is thus a substantial amount of work suggesting that iconicity provides benefits to successful communication and bootstrapping. Such work, however, has tended to either assume that these benefits principally take the form of comprehension benefits or to categorize them broadly as communicative benefits. No work to our knowledge has focused specifically on identifying production benefits in communication.

#### Our Approach.

To investigate the existence of a production benefit from iconicity, we adapted a laboratory language game paradigm devised by Roberts and Clark (2020, 2023). Their study investigated the emergence of dispersion in phonological systems and involved pairs of participants playing a referential communication game in which they took turns to be *Sender* and *Receiver*. The Sender would have a referent (an animal silhouette) selected for them and would have to send a signal to the Receiver to help the Receiver select the same animal silhouette on their screen. The Sender would generate the signal by moving their finger around on a rectangular trackpad; as they did so, colors would appear on their screen based on a mapping between finger position and a continuous underlying colorspace. By holding their finger in place the Sender could select the currently displayed color to send. In Roberts and Clark’s (2020) experiment, iconic signals were certainly possible (animals, after all, are colored in real life) and common, but the iconicity afforded did not make communication trivial. It is not obvious, for instance, that a “bird”, a “butterfly”, or a “dog” immediately suggests one particular color, or at least that any particular color among those available will immediately and unambiguously suggest to the Receiver which animal to select. This was borne out in participants’ behavior and in how well they did at the game. Roberts and Clark (2020) calculated a success index for dyads based on how many referents participants had established reliable signals for in each round; this index was able to capture in one figure both the number of referents they were successfully signaling and how fast they reached that number. It was clear across all conditions that participants found the task challenging and took time to establish reliable signals.

The paradigm we employed for our experiment was based on theirs but involved several differences. First, we designed a colorspace that, for our chosen set of six referents (fruits and vegetables) would allow participants to create signals with a high degree of primary iconicity. Second, we included a condition where the Sender selected not a color but simply a representation of their finger position in a 2-dimensional space—making iconicity very difficult indeed—and a condition in which the Sender selected a color but the Receiver received a finger position. This mismatched condition, or the One-sided iconicity condition, illustrates a strength of the laboratory language game approach—we were able to confine the effects of iconicity to only the Sender to isolate a potential production benefit, something that would be quite difficult in natural language scenarios. Third, we made Sender and Receiver roles fixed in all conditions, rather than having participants take turns; this meant that participants were not aware that the signal the Sender sent might not be the signal that the Receiver received. This was critical to the functionality of the One-sided iconicity condition. Fourth, to provide extra motivation to communicate successfully, we informed participants that the game would end early (but their reward would remain the same) if they developed stable signals for all referents.

### Research Question and Hypothesis

Our research question was as follows: To what extent does iconicity aid bootstrapping when it is available only to the signal producer? We investigated this by manipulating who was exposed to iconic signs. In real-world bootstrapping settings, iconicity is usually available to both senders and receivers of signals (although different individuals might interpret it differently or overlook it; Sehyr & Emmorey, 2019). However, in this study, we used the laboratory language game paradigm to isolate the impact of iconicity, in one condition, to the Sender only. We predicted that there would be a production benefit derived from iconicity that would help dyads communicate more successfully. That is, we predicted that communicative pairs where only the Sender had access to iconicity would perform better than pairs in which neither interlocutor had access to iconicity, even though the former situation involved a misalignment of signals (because Senders saw different signals from Receivers) that the latter did not.

## METHODS

To answer our research question, we designed a dyadic referential communication game in which one member of the dyad, the Sender, generated a signal each round to communicate one of a set of referents (black-and-white images of fruits and vegetables) with another participant, the Receiver. There were three between-subjects conditions; in the *High iconicity condition*, the Sender could generate highly iconic signals (colors), which the Receiver would see. In the *Low iconicity condition*, the Sender could generate only signals with very little iconicity (a dot in a 2-dimensional space), which, again, the Receiver would see. In the *One-sided iconicity condition*, the Sender could generate the same highly iconic signals as in the High iconicity condition, but—unbeknownst to the Sender—the Receiver would see a Low iconicity signal instead. As such, both participants in this condition were misled about the communicative medium available to their partner, leading to a misalignment in the form of the signals. We predicted that dyads would nonetheless communicate more successfully in the One-sided iconicity condition than in the Low iconicity condition. We predicted this despite (a) the misalignment in signals, and (b) signals being no more iconic for the Receiver in the One-sided iconicity condition than in the Low iconicity condition.

### Participants

136 University of Pennsylvania students (10 left-handed), none of whom suffered from color-blindness, participated in 68 dyads for course credit. The rationale for data collection was to gather 40 participants per condition as a minimum and then to continue gathering data until the subject pool closed.

### Materials

#### Physical Equipment and Software.

Participants sat in separate cubicles, from which they could not see each other, each with a computer (a mid-2014 Apple iMac with a 21.5″ screen), running custom-designed software written in Python (Van Rossum & Drake, 1995) and Kivy (Virbel et al., 2011). The software was based on previous software used by Roberts and Clark (2020). To reduce the chance of participants hearing each other, each dyad member wore a pair of noise-canceling headphones (specifically a Sony Wireless Noise Canceling Stereo Headset). Participants with the role of Sender used a wireless multitouch trackpad (a 2012 Apple Magic Trackpad, measuring 13.01 cm by 13.13 cm).

#### Experimental Stimuli.

**Figure 1.**
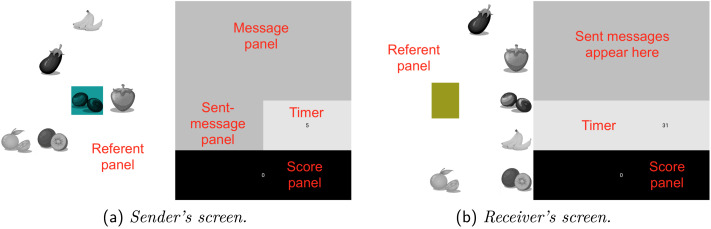
Screenshots of Sender’s and Receiver’s screens. Labels are for clarity and were not shown to participants.

The experiment involved the communication of referents that could be signaled iconically using colors and which could not easily be signaled iconically using a dot positioned in a 2D space. For this we used a set of six black-and-white images of fruits and vegetables selected and adapted from a larger set of originally colored images (Appendix A). The final set consisted of the following items: banana, eggplant, strawberry, blueberry, orange, and kiwi. Images of these as they appeared on screen can be seen in Figure 1.

To establish this final referent set, as well as our colorspace, we conducted a normalization study prior to full piloting. This study took the form of a survey created using Qualtrics (Qualtrics, 2005). 100 University of Pennsylvania students participated individually for course credit and were distributed evenly across four experimental conditions, which differed as follows. In Condition 1, participants were shown a series of 18 black and white pictures, each depicting a different fruit or vegetable. In each case they were asked to select one of 16 color options that they thought would best communicate the fruit/vegetable to another person. Condition 2 worked the same except that, instead of colors, participants were asked to select from a set of 15 *dot positions*, that is, two-dimensional white squares, each of which contained a black dot in a different location. Conditions 3 and 4 were the reverse of Conditions 1 and 2; that is, participants were shown series of colors or dots and asked to select from a set of fruits and vegetables. Appendix B contains sample questions from all four conditions as well as visualizations of results from all conditions, which we analyzed qualitatively in order to select six referents that seemed to have strong associations with particular colors but little association with any particular dot position. For instance, bananas were very reliably associated with the color yellow but showed no consistent association with any specific dot position. Although not directly addressed by our norming study, we also took into account the discriminability of the different fruit images in selecting the final set.

### Procedure

Pairs of participants played a cooperative communication game. At the onset, participants were given instructions (Appendix C) assigning them to either the Sender or Receiver role. They kept, and never swapped, these roles throughout the game. Each participant saw a screen divided into several sections (for the most part, the screen looked similar whether the player was Sender or Receiver; Figure 1). In the left half of the screen—the *referent panel*—the six fruits and vegetables were displayed in a random arrangement. The top right quarter of the screen—the *message panel*—appeared gray by default, but would change depending on the behavior of the Sender. The same was true of a smaller section immediately below it—the *sent-message panel* (see Experimental Conditions section for a description of how the message panel and the sent-message panel worked across conditions). To the right of the sent-message panel, a timer was displayed on a white background. Below this was a score panel displaying the dyad’s joint score against a black background.

The referent panel differed slightly for the Sender and the Receiver. First, the referents were not in the same places (i.e., they were redistributed at random across a 5 × 5 grid separately for each round and participant). Second, no referent was ever in the center of the Receiver’s referent panel; the Sender, on the other hand, always had one referent in the center, against a blue background. This was the *target referent*, which was selected from the full set at random by the server each round. Third, the Receiver (and not the Sender) had a yellowish-green cursor that could be moved around the referent panel by using the arrow keys on the computer keyboard.

The Sender’s task was to convey the target referent to the Receiver by using their trackpad to generate a signal (see Experimental Conditions section). The Receiver’s task, upon receiving this signal, was to move their cursor using the arrow keys on their keyboard to the correct referent and press the return key to select it. Both players would then receive feedback. If the Receiver guessed correctly, the target referent would be highlighted in blue for the Receiver and in yellowish-green for the Sender, and the dyad would score a point, displayed in the score panel for both participants. If the Receiver guessed incorrectly, the target referent would be highlighted in blue and the referent guessed by the Receiver would be highlighted in yellow-green for both participants. No points would be scored in this case. A round lasted a maximum of 30 seconds in total, but would end as soon as the Receiver had selected a referent. If the Receiver did not choose a referent within 30 seconds, the dyad scored no points for that round. Feedback lasted an additional 2 seconds.

Each game would last for 60 minutes in total unless the dyad successfully triggered the winning condition, determined as follows: The Receiver had to have achieved at least 75% success in guessing *each* of the six referents over the previous four rounds in which the referent in question had been the target referent. That is, every time the Receiver guessed correctly, the software checked—for every referent—if at least three out of the four most recent guesses had been correct. If that was the case for all referents, the winning condition had been met. This had the consequence that both positive and negative progress were possible; if a dyad started to do worse at communicating a particular referent, they would move further from satisfying the winning condition. As soon as the winning condition was achieved, however, the game would end. If it was not met, the game would finish at the end of the current round once 60 minutes had passed. Participants were not told the precise details of the winning condition in the instructions. However, to incentivize good performance, they were told that the game would end early—but they would still be paid the full amount—if they did “very well” (Appendix C).

At the start of the experiment, participants played eight practice rounds that differed from the main rounds in three ways: First, to help participants get used to the game, the practice rounds lasted 90 seconds rather than 30 seconds; second, the dyad’s score from these rounds did not carry over into the normal rounds and guesses did not count towards calculating the winning condition; third, participants were reminded of their role at the start of each of the first two rounds. The Sender was told to move a finger around the trackpad and observe the screen, and to hold a finger in the same position for 1 second to send a message (for more detail about signaling, see Experimental Conditions section); otherwise, the Sender was not instructed how to use the signaling medium, but rather had to explore it on their own. After the practice rounds ended, both participants were reminded of the most crucial or complex portions of the instructions (Appendix C).

### Experimental Conditions

There were three between-subjects experimental conditions (summarized in Table 1): *High iconicity*, *One-sided iconicity*, and *Low iconicity*. 22 dyads participated in the High- and Low iconicity conditions each, while 24 dyads participated in the One-sided iconicity condition.^1^ These conditions differed solely in the nature of the signals as they appeared to the Sender and Receiver. Everything else remained the same across conditions.

**Table 1.** Table of conditions

Condition	Sender sees	Receiver sees
High iconicity	Color	Color
Low iconicity	Dot	Dot
One-sided iconicity	Color	Dot

#### High Iconicity.

**Figure 2.**
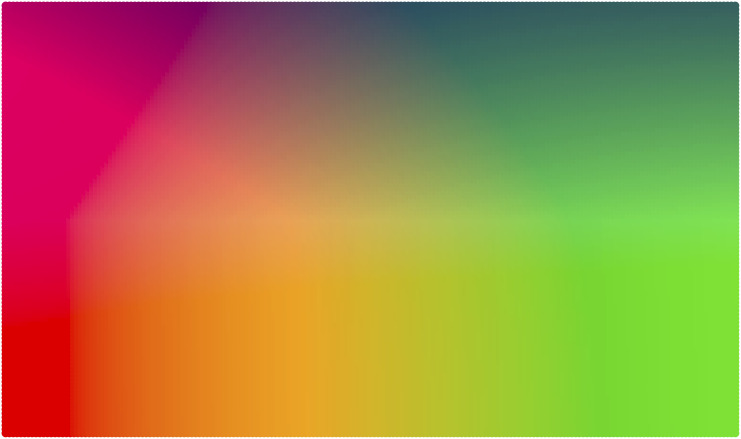
Underlying colorspace for High iconicity and One-sided iconicity conditions.

**Figure 3.**
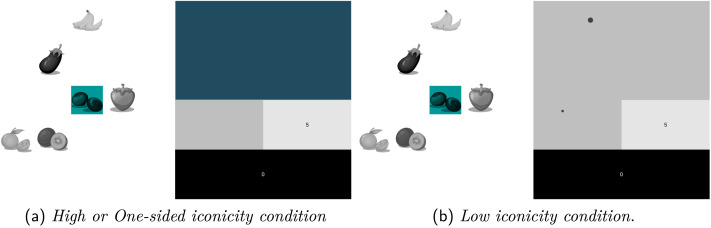
Example screenshots of Sender’s screen in different conditions.

In the High iconicity condition, the Sender’s finger position on the trackpad would correspond dynamically to a color displayed in the message panel on the top right of the Sender’s screen (Figure 3a); this would update continuously based on finger position (the underlying colorspace is displayed in Figure 2). For instance, if the Sender positioned their finger in the bottom-left corner of the trackpad, the message panel would turn red; if they moved it along the bottom edge of the trackpad the color would change to become more orange, then more yellow, and eventually more green (Figure 2). If the Sender took their finger off the trackpad or touched the trackpad with more than one finger, the message panel would return to its gray default appearance. If the Sender held their finger in place on the trackpad for at least 1 second, the currently displayed color would appear on both the Receiver’s message panel and the Sender’s sent-message panel. This was the only means by which the Sender could send information to the Receiver.

#### Low Iconicity.

In the Low iconicity condition, the Sender was, as in the High iconicity condition, instructed to move their finger around their trackpad to attempt to communicate the target referent to the Receiver. However, instead of their finger position on the trackpad corresponding to a color, it corresponded to a circular black dot positioned on the message panel (Figure 3b). For instance, if the Sender positioned their finger in the bottom-left corner of the trackpad, the dot would appear in the bottom-left corner of the Sender’s message panel; if they moved it along the bottom edge of the trackpad, the dot would move further right accordingly. As in the High iconicity condition, if the Sender took their finger off the trackpad or touched the trackpad with more than one finger, the message panel would return to its gray default appearance. If the Sender held their finger in place on the trackpad for at least 1 second the current dot would appear on both the Receiver’s message panel and on the Sender’s sent-message panel. This was the only means by which the Sender could send information to the Receiver.

#### One-Sided Iconicity.

In the One-sided iconicity condition, the Sender’s experience was precisely as in the High iconicity condition (i.e., their finger position corresponded to a color). However, when the Sender held their finger in place on the trackpad for at least 1 second in order to send the color, the Receiver would see not the color, but instead a dot corresponding to the finger position of the Sender (as the Receiver in the Low iconicity condition would). The Sender, by contrast, would see the color they selected in the sent-message panel exactly as in the High iconicity condition. Neither the Sender nor the Receiver was informed of this communicative mismatch.

### Colorspace Design

Figure 2 shows the underlying colorspace used in the High iconicity and One-sided iconicity conditions. This was based on a CIELAB colorspace (Luo, 2023), which involves three values: L* controlling perceptual lightness (with black at 0 and white at 100), a* controlling the green-red spectrum (with negative numbers towards green and positive numbers towards red), and b* controlling the blue-yellow spectrum (with negative numbers towards blue and positive numbers towards yellow). The a* and b* variables were computed as follows, where x and y are the x- and y-coordinates of the finger position and w is the length of the x axis.

$${\text{a}}^{*}=-128+\left(\left|\frac{\text{w}-\text{x}}{\text{w}}\right|\times 256\right)$$ (1)

$${\text{b}}^{*}=-128+\left(\left|\frac{\text{w}-\text{y}}{\text{w}}\right|\times 256\right)$$ (2)

L* was based on finger position along the y axis following a conditional rule. If the finger was located in the precise center of the y axis or in the bottom half of the trackpad, L* was set to 75. Otherwise, L* was calculated using the formula 120 − (90 × $\frac{y}{h}$), where *y* is the y-coordinate of the finger position and *h* is the length of the y axis.

Participants were not given details about the underlying colorspace but were instructed to explore this for themselves (Appendix C).

## RESULTS

In this section we begin by presenting a measure of success specific to the Sender: signal stability. In this measure, the One-sided iconicity condition patterns similarly to that of High iconicity, suggesting the presence of a production benefit from iconicity. Then, we take a bird’s-eye view of the success of dyads as a whole, reporting mean accuracy, number of rounds, and time-to-winning for the three conditions. We also present the mean success index for each condition, based on the measure originally used by Roberts and Clark (2020), with modifications made due to differences in design. For these measures, contrary to expectations, the One-sided iconicity condition patterned more like the Low iconicity than the High iconicity condition; the difference in producer-side signal stability does not correspond to an equivalent difference in dyadic success, broadly construed. However, we finally present a round-by-round analysis which suggests that One-sided iconicity in fact provided a clear head-start to dyads, allowing them to overtake dyads in the Low iconicity condition. However, consistent with the coarser measures of dyadic success, establishing signals for the full set of six referents still proved difficult in the One-sided iconicity condition. This difficulty was almost certainly due to the lack of a comprehension benefit. That is, the lack of semantic transparency likely heightened processing costs and increased memory load on the part of the Receiver, preventing dyads in the One-sided iconicity condition from achieving similar success to those in the High iconicity condition.

Data were parsed using Python (Van Rossum & Drake, 1995). Analyses were conducted using the R Statistical environment (R Core Team, 2014); linear models were run using the lme4 and lmerTest libraries (Bates et al., 2015; Kuznetsova et al., 2017), with the performance package used to calculate *r*^2^ (Lüdecke et al., 2021). Plots were created using ggplot2 (Wickham, 2016). Residual and QQ-plots are provided in Appendix E. Data and analysis scripts are available at https://doi.org/10.17605/OSF.IO/MCXF7.

### Sender-Side Analysis

**Figure 4.**
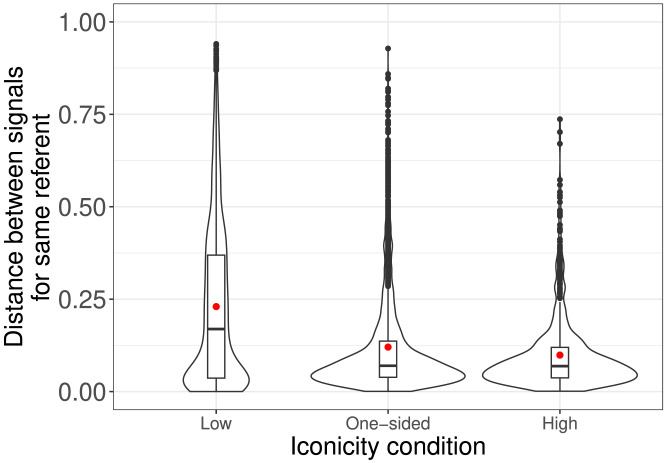
Violin plots of distances between successive signals for the same referent by condition, overlaid with boxplots. Practice rounds are included. Red dot indicates mean.

If Senders in the One-sided iconicity (and High iconicity) conditions indeed experienced a production benefit from iconicity, this would be likely to manifest in *signal stability*. As argued earlier, if iconic forms are easier to remember, they are more likely to be reused; even if they are forgotten, they are more likely to be generated again. Both of these factors should lead to high signal stability. We operationalized stability by measuring, for each signal in each game, the distance between the Sender’s finger position on the pad and their finger position for the previous signal for the same referent (Figure 4). Lower distances mean Senders were more consistently hitting the same spot. A linear mixed-effects model with distance as dependent variable, condition as independent variable (Low iconicity as reference level), and random intercepts for dyad and referent revealed an effect for both One-sided iconicity, *β* = −0.07, *SE* = 0.02, *t*(65.85) = −3.1, *p* = 0.0029 and High iconicity, *β* = −0.09, *SE* = 0.02, *t*(71.82) = −3.8, *p* < 0.001; *r*^2^ = 0.19. The same model with High iconicity as the reference level indicated no difference between that and One-sided iconicity: *β* = −0.02, *SE* = 0.02, *t*(71.83) = −0.9, *p* = 0.37.

### Dyadic Analysis

**Figure 5.**
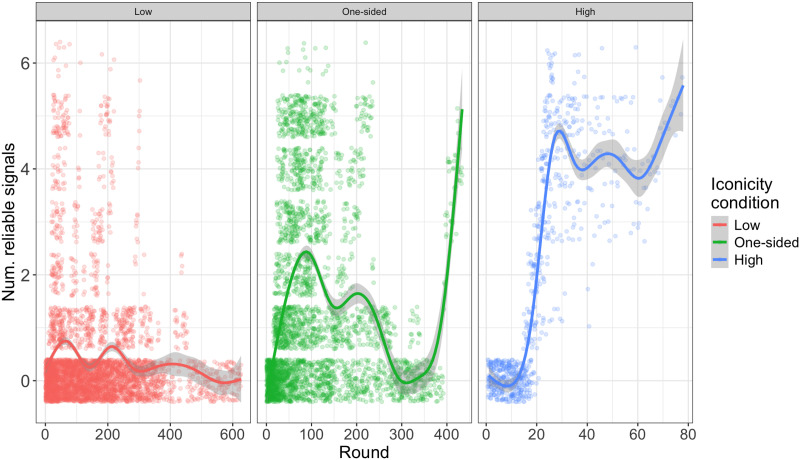
Number of reliable signals established by round, faceted by condition. Each jittered dot indicates how many signals a given dyad had established in that round (provided they were still playing). Note that *x* axis limits differ significantly between conditions because of the difference in number of rounds (compare Figure 6).

As discussed above, dyads where the Sender had access to iconicity experienced greater signal stability than those that did not. What were the consequences of this for participants’ success over the course of the game? Figure 5 shows how many signals dyads had established on each round before they completed the game. What stands out in this plot is both that there was variation between conditions in how fast participants completed the game and that there was variation in how many referents they managed to establish signals for. In the following sections we present several different measures designed to get at this.

#### Time Taken.

**Figure 6.**
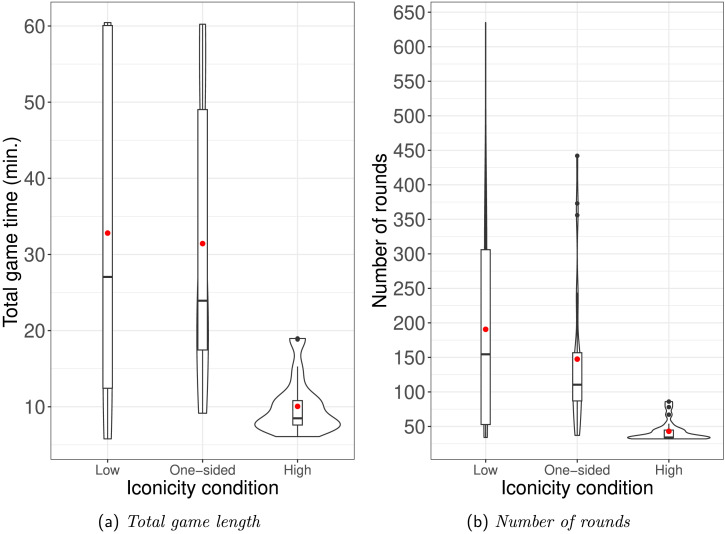
Violin plots, overlaid by boxplots, of game length and number of rounds by condition. Red dot indicates mean.

We examined how long dyads took to establish successful communication, in terms of both time and number of rounds. As mentioned earlier, the game ended immediately once dyads had established signals for all six referents. If dyads did not manage this, it continued for a full hour. Figure 6 shows the total game time and total number of rounds by condition. As can be seen in Figure 6a, dyads in the High iconicity condition completed the game much faster than dyads in other conditions—no dyad took more than 19 minutes, and the mean was 10 minutes, a third of the mean time taken in the other two conditions! In other words, dyads in the High iconicity condition did much better at the communication game than dyads in either of the other conditions. This is reflected in the results of a linear model with game length as dependent variable and condition as independent variable (Low iconicity as reference level): *β* = −1365.55, *SE* = 304.49, *t* = −4.49, *p* < 0.001. However, there was no such difference between Low-iconicity and One-sided iconicity: *β* = −82.83, *SE* = 298.08, *t* = −0.28, *p* = 0.78.

#### Accuracy.

We defined *accuracy* as the Receiver’s percentage success at guessing the correct referent in different rounds. A linear model with accuracy as dependent variable and condition as independent variable (Low iconicity as reference level) revealed an effect for High iconicity, *β* = 0.42, *SE* = 0.05, *t* = 8.78, *p* < 0.001, and a more modest effect for One-sided iconicity: *β* = 0.1, *SE* = 0.05, *t* = 2.12, *p* = 0.038; *r*^2^ = 0.57.

#### Success Index.

We also calculated a success index for each dyad, following the method employed by Roberts and Clark (2020), which was designed to take into account the number of referents that participants established reliable signals for and how fast they did so. By “reliable signal” we mean that for at least three out of the four most recent occasions the referent in question was a target, the dyad communicated it successfully. (This is the same criterion as used within the game for the winning condition, as described in the Procedure section.) The logic here is that guessing the right or wrong referent could happen accidentally on a particular attempt, so several turns need to be taken into account to feel confident. Furthermore, a dyad who establishes reliable signals for all referents within (e.g.) 100 rounds is doing better than one who takes 200 rounds, so our metric also took this into account. First, for each dyad, we identified how many signals they had established in each round of the game (not including practice rounds). Then we calculated a success index as ($\sum_{1}^{n_{r}}$ *s*)/6*n*_*r*_, where *n*_*r*_ is the number of rounds and the numerator is thus a cumulative count of *s*, the number of successfully established signals for referents in a given round (with six being the maximum possible given the number of referents).^2^

Our success index differed from that of Roberts and Clark (2020) in that we included practice rounds and “dummy rounds” in calculating it. In earlier work, practice rounds were not included in success index calculations because they differed from other rounds in several respects (see Procedure section). In our experiment, however, the speed with which dyads in the High iconicity condition established signals meant that, for this condition, a good deal of the progress in establishing signals occurred during the practice rounds, which consequently took up a comparatively substantial proportion of their total rounds (e.g., one dyad won the game in 32 rounds, meaning that practice rounds consisted of a quarter of the total). In earlier studies, the communication game was not only harder than our High iconicity condition, but dyads always played for the full time however well they did. (In fact, Roberts & Clark, 2020 found that including or excluding practice rounds made little difference to the pattern of results.)

As noted, we included practice rounds in part due to variation in game completion time among dyads (Figure 6a); however, incorporating these does not fully correct for how differences in trial length affect the success index. We solved this by adding “dummy rounds” (with all six referents established) for every dyad who finished in under an hour. This simulated what would have happened if dyads had been made to continue playing for a full hour even if they had established signals for all referents. We did this by calculating the mean length of the final four rounds of each game and then, based on this, calculating an estimate of how many extra rounds the dyad in question would have played if they had continued for a full hour. We then calculated the success index with these rounds included. Appendix D presents success indices without practice and without dummy rounds for comparison purposes.

**Figure 7.**
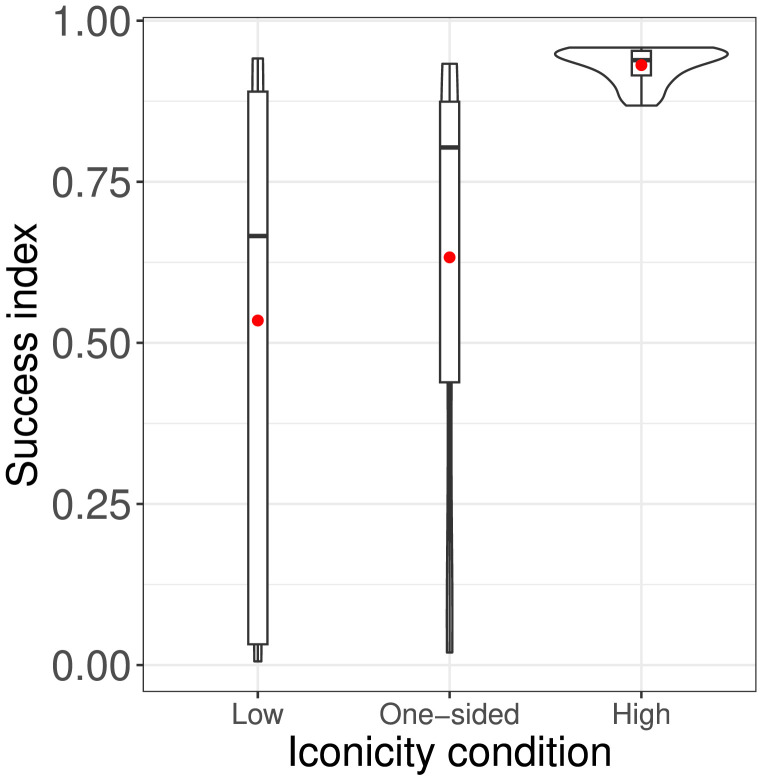
Violin plots of success index by condition, overlaid with boxplots. Practice rounds and dummy rounds are included. Red dot indicates mean.

Figure 7 shows success index by condition. As can be seen, participants did best in the High iconicity condition. A linear model with success index as the dependent variable and iconicity condition as the independent variable (with Low iconicity as the reference level) found that success in the High iconicity condition was significantly higher than for the Low iconicity condition, *β* = 0.396, *SE* = 0.09, *t* = 4.36, *p* < 0.001. However, the success index for the One-sided iconicity condition was not—contrary to expectation—significantly higher than in the Low iconicity condition: *β* = 0.098, *SE* = 0.09, *t* = 1.1, *p* = 0.275; *r*^2^ = 0.24.

### Round-by-Round Analysis

The results reported above do not mean that the greater signal stability in the One-sided iconicity condition did not help dyads establish signals. Importantly, while they did not complete the game anywhere near as fast as dyads in the High iconicity condition, they do seem to have found it easier than dyads in the Low iconicity condition to start establishing reliable signals. We can investigate this by taking a closer round-by-round look at the basic data (without dummy rounds but including practice rounds), as displayed in Figure 5.

As this data contained a number of zeros (i.e., trials with no referents), we performed two analyses, having first coded trials with no referents as zero and trials with at least one referent as one. In our first analysis, designed to investigate whether there was an effect of condition on establishing signals at all, we ran a binomial linear mixed effects model with referent presence as the dependent variable, and round number (scaled) and condition (with Low iconicity as the reference level) as independent variables, along with their interaction, and random intercepts for dyads. There was a main effect of round number, *β* = 0.83, *SE* = 0.07, *z* = 12.19, *p* < 0.001, a main effect of condition for One-sided iconicity, *β* = 1.06, *SE* = 0.49, *z* = 2.17, *p* = 0.03, and for High-iconicity, *β* = 160.1, *SE* = 18.45, *z* = 8.68, *p* < 0.001, as well as an interaction between round number and condition: *β* = −0.496, *SE* = 0.09, *z* = −5.26, *p* < 0.001 (for One-sided iconicity); *β* = 175.11, *SE* = 20.35, *z* = 8.61, *p* < 0.001; *r*^2^ = 0.95.

We then ran a negative binomial linear mixed effects model on the non-zero data with number of reliable signals (as defined for the purpose of the success index, but excluding zeros) as the dependent variable, and round number (scaled) and condition (with Low iconicity as the reference level) as independent variables, along with their interaction, and random intercepts for dyads. This revealed a main effect of condition for One-sided iconicity, *β* = 0.40, *SE* = 0.16, *z* = 2.47, *p* = 0.01, and for High iconicity, *β* = 0.40, *SE* = 0.25, *z* = 10.50, *p* < 0.001 as well as an interaction between round number and condition: *β* = 0.20, *SE* = 0.05, *z* = 4.02, *p* < 0.001 (for One-sided iconicity); *β* = 2.03, *SE* = 0.22, *z* = 9.08, *p* < 0.001 (for High iconicity); *r*^2^ = 0.61. These results support the view that dyads in both these conditions experienced a head-start in establishing referents. This was substantial in the High-iconicity condition; while it was more modest in the One-sided iconicity condition, it seems to have made a difference.

**Figure 8.**
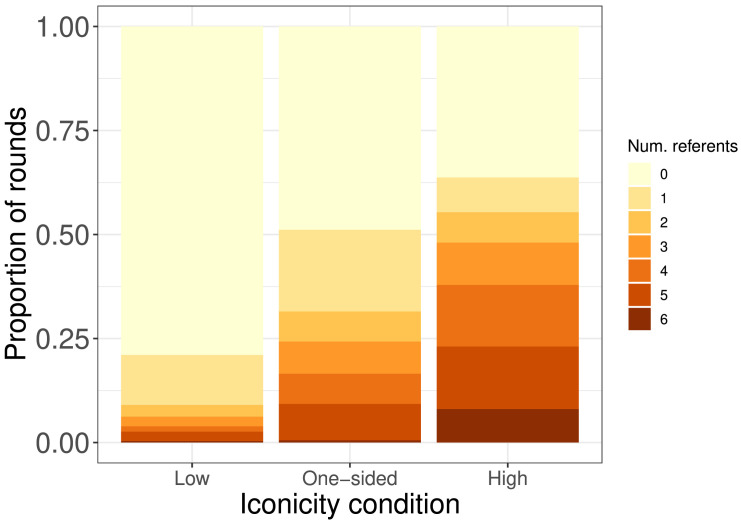
Proportion of rounds with each signal set size across all dyads, organized by condition (practice rounds included).

More particularly, they seem to have experienced an advantage in getting communication off the ground. Figure 8 shows, for each condition, the proportion of rounds dyads spent with different numbers of established signals. (Practice rounds, but not dummy rounds, are included.) As can be seen, most rounds (>75%) in the Low iconicity condition were spent having established no reliable signals at all. This was true for less than 48% of rounds in the other two conditions, and in this respect the One-sided condition looks more like the High iconicity condition, the main difference being that dyads in the former spent more time (21% vs. 8%) with only one referent. Notably, however, dyads in the One-sided iconicity condition spent more rounds (31.8%) with established signals for at least half of the referents than dyads in the Low iconicity condition spent with any established signals at all (21.7%).

Dyads in the One-sided iconicity condition thus seem to have experienced a head-start. However, as suggested by the total game time for the One-sided iconicity condition (Figure 6a), they did not find it as easy to establish the full set of six signals that would have allowed them to complete the game. This makes sense, as Receivers in the One-sided iconicity condition did not have access to the comprehension benefit derived from iconicity, while those in the High iconicity condition did. Our results do not allow us to determine what the exact make-up of this comprehension benefit is, but one potential contributing factor is that iconicity reduces challenges of memory load for the Receiver. That is, while greater consistency in the One-sided iconicity condition helped Receivers memorize more arbitrary signals than in the Low iconicity condition, memorizing the full set of six presented a challenge.

## FOLLOW-UP EXPERIMENT

**Figure 9.**
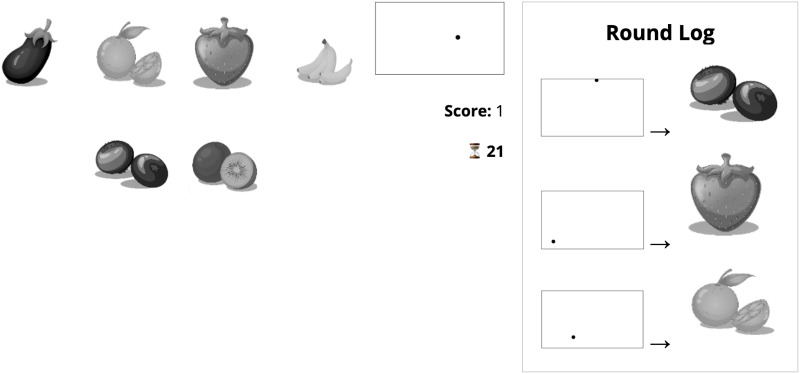
Screenshot from follow-up experiment.

To investigate whether memory load might help explain the difference between the One-sided and High iconicity conditions, we conducted a follow-up experiment in which individual online participants played the role of Receivers with automatically presented signals. The interface resembled the interface in the original experiment (Figure 9). To investigate the role of memory, we manipulated the presence or absence of a log, visible on the right of the screen, displaying signals for some of the referents. Specifically, for four of the referents, the first signal presented for that referent (i.e., the stimulus shown in the first round in which that referent was the target) would be added to the log after the participant’s guess as a reminder in future rounds of what referent that signal referred to. We also manipulated whether the signals were color- or dot-based, as in the original experiment. These two independent variables were crossed in a between-subjects 2 × 2 design.

### Methods

#### Participants.

80 participants were recruited using the online crowdsourcing platform Prolific (Palan & Schitter, 2018). Condition was initially assigned randomly to each participant. After we had gathered 58 trials, we examined the distribution and gathered trials for each condition manually, one-by-one, to ensure equal distribution of participants across conditions.

#### Procedure.

Each participant took the role of Receiver. There were no participant Senders. In each “round” a participant would be presented with a signal, which (depending on condition) would either be a color or a dot, as in the original experiment. The signals used in this experiment were collected from a single trial in the High iconicity condition of the original experiment, chosen at random. For each referent, for the first three rounds in which that referent was the target in the randomly-chosen High iconicity trial, we recorded the x and y coordinates chosen by the Sender. We used three signals for each referent in order to replicate the variation and noise observed in trials of the original experiment (which should be expected to be relevant to memory load). We used these 18 coordinate pairs (i.e., three coordinate pairs for each of the six referents) to create 18 color signals and 18 dot signals to be shown to participants in the present experiment, depending on condition. These signals were not presented in any particular order to participants; the signal displayed in any given round was chosen randomly.

Figure 9 shows an example screen with one of the dot signals visible. After receiving the signal, the participant would have 30 seconds to select which referent they thought was being signaled to them by clicking on that referent. They then received feedback, followed by a new round. The feedback was as follows: If the participant guessed correctly, they received a “Correct!” message; if the participant guessed incorrectly, they received an “Incorrect!” message and were informed of the correct referent. Participants completed as many rounds as they could in 15 minutes, not including time spent reading the consent form and instructions. In conditions with a log, there was a space on the right of the screen that would show a correct signal for four referents. These were not shown at the beginning of the game. Rather, four referents were randomly pre-selected and, for each of them, at the end of the first turn in which that referent was the target, the signal displayed in that round would appear in the log, next to a picture of the referent. The signal would remain in the log for the remainder of the experiment and would not be updated in any way.

### Results

**Figure 10.**
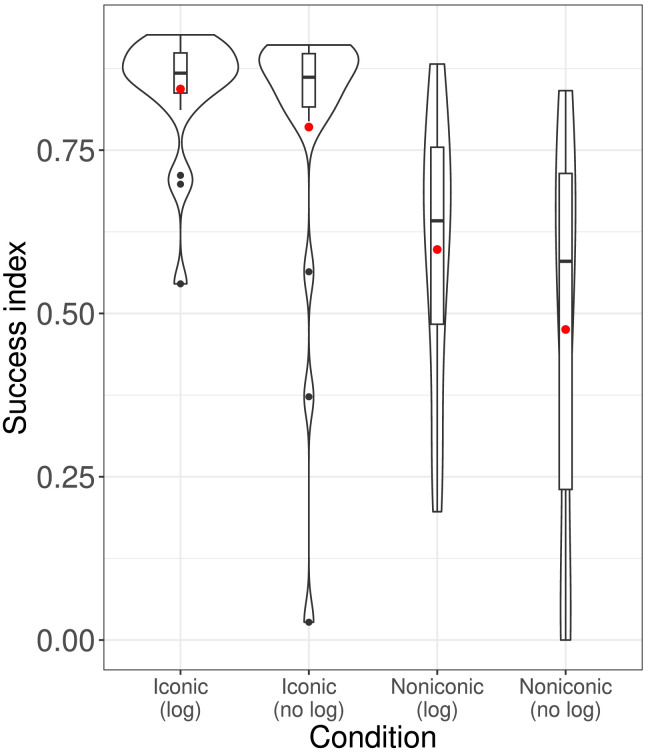
Success indices for follow-up experiment.

We measured the success index for each participant as in the original experiment. Because the experiment was the same length (15 min.) for every participant, we did not need to add dummy rounds. Figure 10 shows the results. There was a difference between signal type (color vs. dot): *β* = 0.31, *SE* = 0.07, *t* = 4.5, *p* < 0.001. However, there was no significant effect of having the log, *β* = 0.12, *SE* = 0.07, *t* = 1.78, *p* = 0.08, and no interaction: *β* = −0.06, *SE* = 0.097, *t* = −0.66, *p* = 0.51.

### Discussion

The results of the follow-up experiment replicate the effect of iconicity helping Receivers identify referents. (Since there was no Sender, and we did not manipulate signal consistency, we cannot tell if we replicated the difference between the original One-sided and Low iconicity conditions.) Somewhat surprisingly, there was no effect of the memory log. It is possible that the experiment was underpowered. Mean success was numerically higher in conditions with a log than in conditions without one, and with a larger sample an effect might well be detected. However, it is notable that success in both dot conditions was significantly lower than in either color condition. There are several possible reasons for this. Some concern artifacts of the experiment. To make use of the log, participants have to actively compare the presented signal (in one part of the screen) with the signals in the log (in another part of the screen). That adds an extra complication to referent selection that is not present if participants are instead relying on an iconic relationship or even on their own memory. Furthermore, the presence of noise in the signal means that the presented signal was often not identical to the log signal for the same referent, which may in some cases have encouraged participants to make the wrong choice. It is also worth noting that participants were only shown four signals in the log. We did this on the basis that memory load should increase as the number of items to recall increases, and also to avoid complicating the visual information. However, this did mean that participants still had to store two arbitrary signals in memory without an aid, an extra step compared with participants in the color conditions, who might store signals in memory, but could also rely on the iconic relation each time instead.

Another possibility that is not specific to the experimental design is that the difference between the High and One-sided iconicity conditions was not (or at least not mainly) due to memory constraints. While it would be surprising if memory load played no role, it is reasonable to the assume that there are also other factors deriving from the difference in iconicity between signal types. These might include processing advantages, not to mention the obvious role played by the semantic transparency of iconic forms. Further studies could employ an approach similar to our follow-up experiment to investigate the individual impact of these other potential advantages.

## GENERAL DISCUSSION

We conducted an experiment to investigate whether long-observed benefits of iconicity in bootstrapping a communication system are reducible to comprehension benefits for the signal receiver, or if communicators also gain from production benefits afforded to the sender producing the signals. The experiment involved a Sender communicating different fruit and vegetable referents to a Receiver by moving their finger around on a trackpad to generate signals. There were three conditions. In the High iconicity condition the signals consisted of colors, which both participants saw and which afforded a high level of iconicity. In the Low iconicity condition each signal consisted of a dot representing the Sender’s finger position on the trackpad, and which afforded very low iconicity. In the One-sided iconicity condition the Sender produced—and saw—colors, but the Receiver saw the sender’s finger position on the trackpad instead. Participants were unaware of this difference in experience.

Dyads in the High iconicity condition were very successful and fast at establishing a communication system, while dyads in the Low iconicity condition were the least successful, being less accurate than in either of the other two conditions, and also the slowest to establish communication systems. The One-sided iconicity condition was the most interesting. There does in fact seem to have been a production benefit, and this gave dyads in the One-sided iconicity condition a head-start (relative to those in Low iconicity) in bootstrapping signals: Most rounds in this condition—unlike in the Low iconicity condition—were spent with at least one signal established. In fact, nearly a third of rounds in this condition were spent with signals established for half the referents. This was likely at least in part due to the greater signal stability observed in both the High and One-sided iconicity conditions. Getting to all six referents proved challenging, however, due to the absence of the comprehension benefit provided by iconicity. Memory load may play some role in this, but other factors almost certainly also influence this benefit, as demonstrated in our follow-up experiment. In other words, despite the production benefit, the overall success of the dyads was still limited by the lack of a comprehension benefit of the kind provided by iconicity.

Our findings not only shed light on the motivations for iconicity in cases of newly emerging communication systems in the real world, in which the comprehension benefits are evident and hard to separate from production benefits; they also have implications for where we might expect iconicity to occur. If our results do indeed indicate production benefits from iconicity, this suggests that we should not expect iconicity to be restricted to contexts in which there is a clear audience benefit. Rather, we should likely expect iconicity to be a feature of coining new referring expressions, even when aiding comprehension is not a serious concern—such as in talking to oneself, for instance. It seems unlikely, furthermore, that the survival of sound symbolic forms in many vocal languages in the form of onomatopoeia and ideophones has solely to do with ensuring that the expressions in question are understood.

It is important to note that, while our study was designed to separate the role of iconicity for Senders from its role for Receivers, it was also designed to investigate it in the context of interacting pairs, in which members are inherently not independent from each other. This is necessarily reflected in our (joint) success measure. However, it is also manifested in the stability measure displayed in Figure 4. While this is essentially a purely Sender-side measure, the behavior measured is inevitably somewhat influenced and constrained by the performance of receivers. For instance, Receiver unreliability in selecting the correct referent might be expected to lead Senders to vary their signals more than they might otherwise do, such that our study may in fact underestimate the benefits of iconicity to Senders. Our results for this measure (in particular, the lack of an effect for the One-sided vs. High-iconicity conditions) suggest that this might not have been the case. However, it is perhaps worth noting that the mean distance and the variability were a little higher in One-sided iconicity, which in a larger scale study might play more of a role. In future work it would be important to conduct more single-player experiments, as in our follow-up experiment, allowing us to more fully separate the two roles.

On the other hand, it is also worth noting that the majority of studies of this kind involve both participants switching between playing the roles of Sender and Receiver (e.g., Fay et al., 2010; Galantucci, 2005; Roberts & Clark, 2020), a more ecologically valid pattern than in our study. Having fixed Sender and Receiver roles served an important purpose for our design but also created a somewhat odd dynamic, from the point of view of human interaction, in which communication was entirely asymmetric. In future work it would be worth exploring ways around such stark asymmetry.

On a related note, these results also shine an interesting light on the question of alignment in communication. It is often assumed that successful communication relies on alignment between interlocutors at various levels (e.g., Garrod & Pickering, 2009; Poesio & Rieser, 2010). This is often taken to imply close equivalence in representations of both signals and referents. Pickering and Garrod (2004), for instance, discuss priming “at each level of linguistic representation, by percolation between the levels so that alignment at one level enhances alignment at other levels” (p. 175). In the One-sided iconicity condition, however, alignment at all levels was not possible. Not only that, but the instructions they received (Appendix C) strongly implied that the signals the Sender saw were the same as the signals the Receiver saw. They nonetheless performed better than participants in the Low iconicity condition, who were highly aligned. This is not to suggest that alignment is not important in communication, or even that some kinds of alignment—such as the alignment in signal form under discussion here—do not matter at all. Rather, it seems to point at the fact that consistency of mapping is more important than shared representations. Indeed, Pickering and Garrod (2004) discussed the “very interesting issue when alignment at one level conflicts with alignment at another”, giving body-relative directions (e.g., left and right) as an example (pp. 189–190). It is also worth noting that, in our study, the mismatch in representation always co-occurred with a mismatch in iconicity. It would be interesting in future work to disentangle the two factors and look at the role of similarly iconic signals that involve different representations (e.g., such as signals that are iconic in different ways, or in different modalities).

This study also brought us insight with regard to analysis. We initially planned to apply Roberts and Clark’s (2020) success-index metric without including practice or dummy rounds. Through conducting this analysis and paying attention to our data we realized that there was an important difference between our data and the data the metric had previously been used on (viz that we ended the game early by design in several trials). By comparing the results of our first analysis approach with subsequent analyses (see Appendix D) we realized that the discrepancy indicated a more nuanced, interesting, and enlightening narrative than was initially apparent. We would not have realized this had we not examined our data and questioned our initial analysis to the extent that we did.

It is important to note that this game involved a somewhat unusual and non-linguistic communication task. This is now a well-established and relatively widely used approach (Nölle & Galantucci, 2022) and the particular communication system was taken from an established paradigm (Roberts & Clark, 2020, 2023). Indeed, the use of the laboratory language game approach was central to our investigation, as it allowed us to artificially restrict the effects of iconicity to the Sender in the One-sided iconicity condition. However, it would be interesting in future work to investigate whether our results are to any extent influenced by the unfamiliarity of the communication medium or the modality. One possibility is that the challenge of engaging with a novel communication medium might have increased participants’ cognitive load and made the task of memorizing arbitrary signals (for Receivers in the One-sided iconicity condition and for both members of the dyad in the Low iconicity condition) particularly hard.

Future work could also expand beyond the extremely restricted referent set—of six fruits—employed in this experiment. This was kept simple for the sake of not over-complicating our model of communication; however, in reality, meaning is evidently much more complex than this, and the kinds of contextual contrast that communicators must make are a good deal more complex than in our study. In fact, not only did participants communicate only about the same set of six items, but the communicative context always had the same set of items present. Future studies could vary this to take into account the immediate referential context in a more ecologically valid way that is likely to have consequences for the communicative strategies (including patterns of iconicity) employed. In fact, several existing laboratory-language studies provide inspiration on this front (Silvey et al., 2015; Winters et al., 2018; Winters & Morin, 2019).

Such limitations aside, however, we consider that this study shines a nuanced light on the role of iconicity in the bootstrapping of signals and communication systems, and the basis of the communicative benefit it provides, while also contributing to research on the role of alignment in communication. It suggests that iconicity gives communicators a head-start in bootstrapping a signal system even if the audience doesn’t know it’s there.

## ACKNOWLEDGMENTS

We are grateful to several members of the Cultural Evolution of Language Lab for their feedback on study design and assistance with data collection, and to Kathryn Schuler for the use of her server for running the follow-up study.

## FUNDING INFORMATION

This work began as a student summer project supported by the University of Pennsylvania Undergraduate Research Mentoring Program (PURM). The main experiment was partly supported by a National Science Foundation award (1946882) and the follow-up experiment was funded by a Penn University Research Fund award.

## AUTHOR CONTRIBUTIONS

All authors contributed equally to designing the study, analyzing the data, and writing the paper. Data collection was led by RS and conducted by RS and PK, using software written by GR. Follow-up study software was written by PK with assistance from RS.

## DATA AVAILABILITY STATEMENT

The data that support the findings of this study, along with scripts used to analyze the data, are openly available in an Open Science Foundation repository at https://doi.org/10.17605/OSF.IO/MCXF7.

## Notes

^1^ Owing to an oversight, the One-sided iconicity condition contained two extra dyads.^2^ As noted by Roberts and Clark (2020), no dyad could actually score 1, as that would require them to have successfully communicated every referent several times before the start of the game. Correcting for this would have needlessly complicated an index intended as a relative measure.

## Figures and Tables

**Figure A1.**
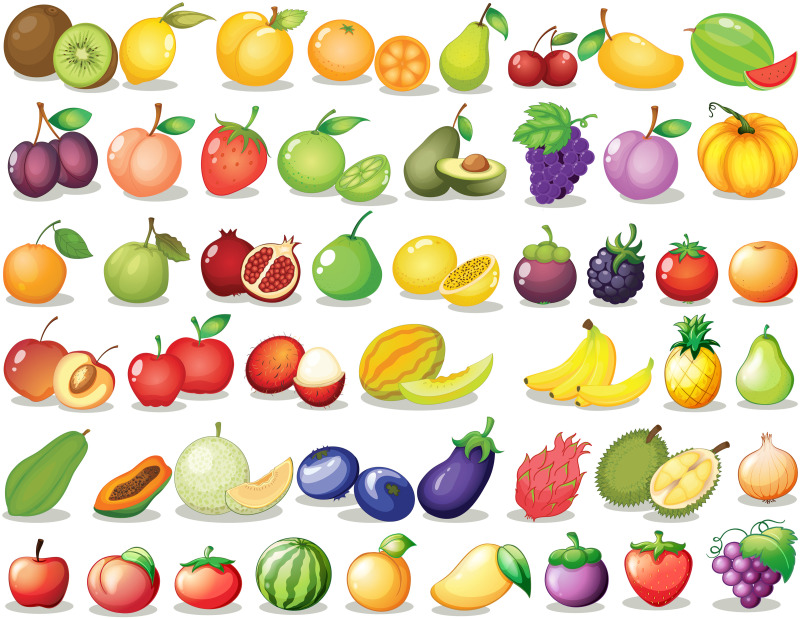
Original image of fruit/vegetable referents. These were cropped and converted to black-and-white for use in the normalization study, from which six were selected for the experiment.

**Figure B1.**
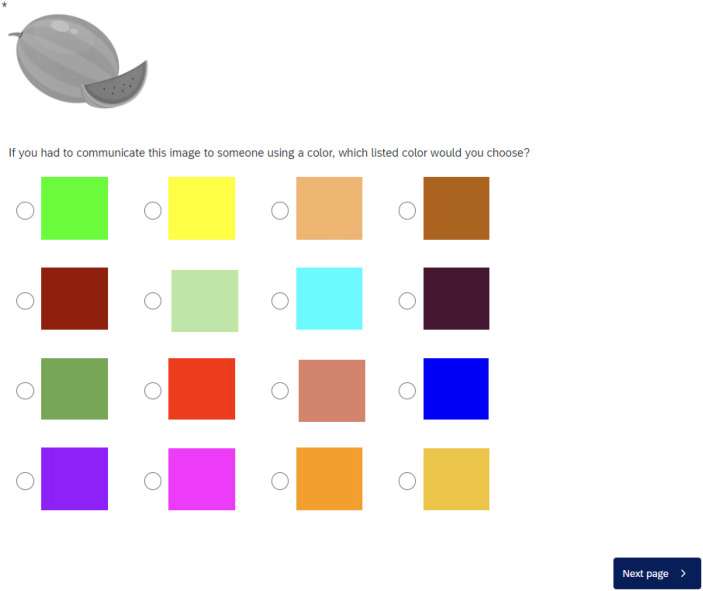
A sample question from Condition 1. The sixteen color options are displayed—these were chosen to cover as much of the color spectrum as possible.

**Figure B2.**
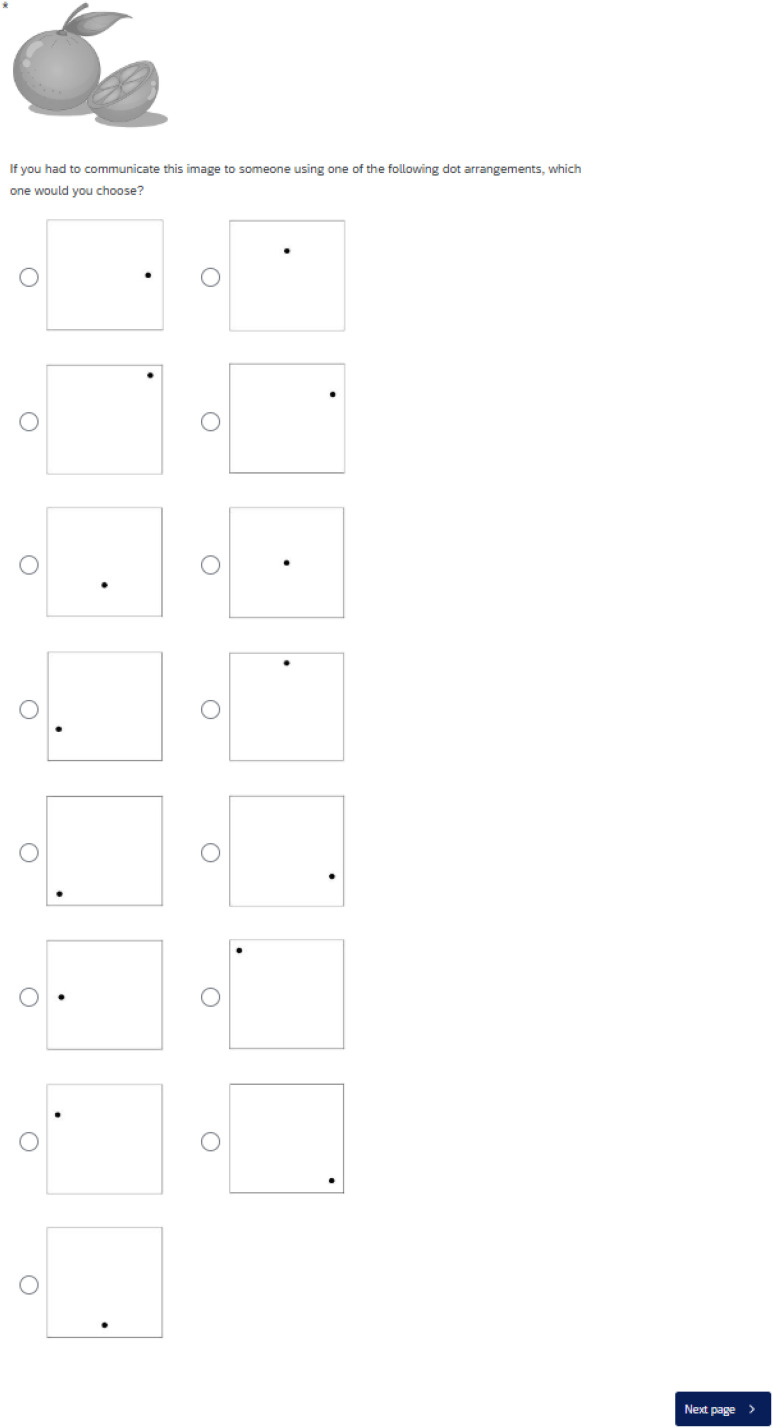
A sample question from Condition 2. The fifteen dot positions are displayed—they are distributed evenly across the square with five rows and three columns.

**Figure B3.**
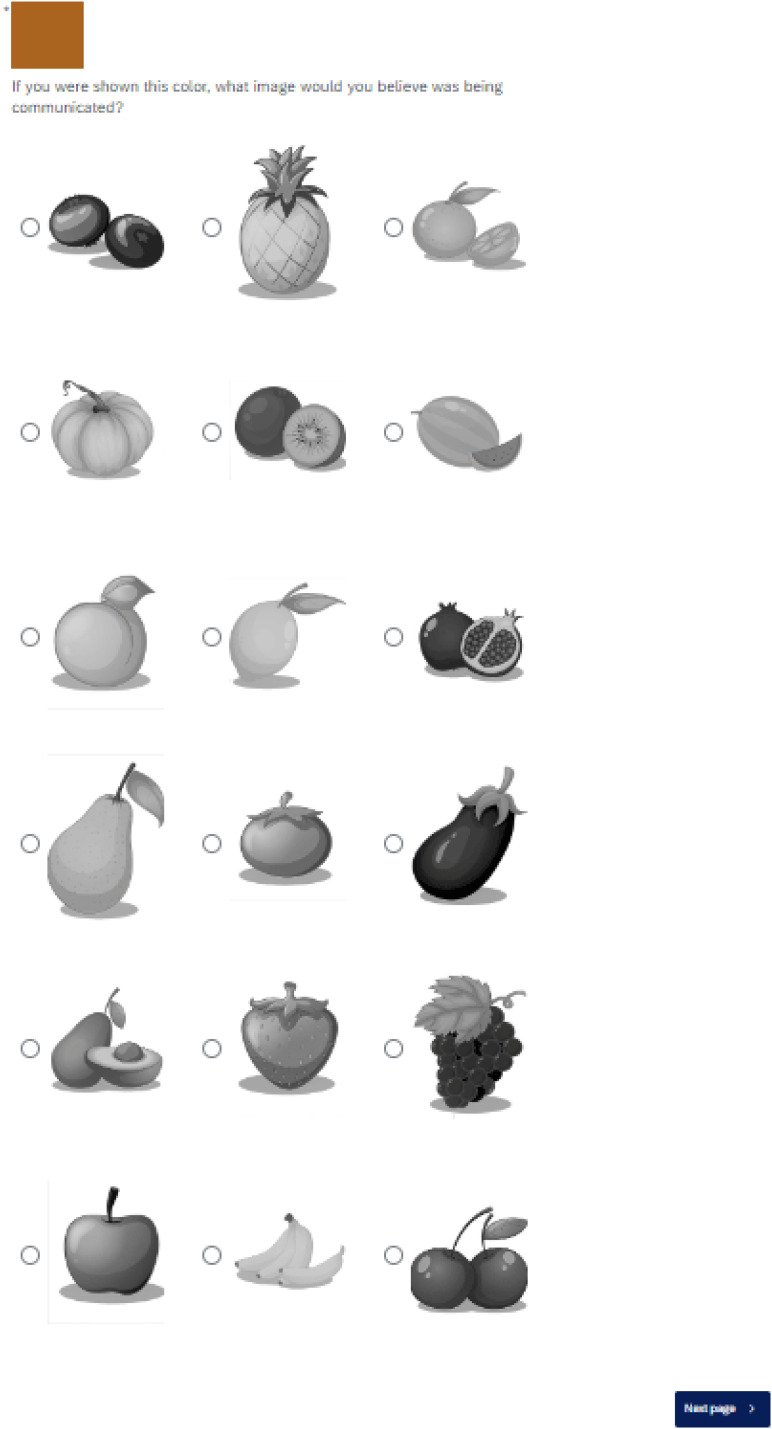
A sample question from Condition 3. The eighteen fruits/vegetables are displayed.

**Figure B4.**
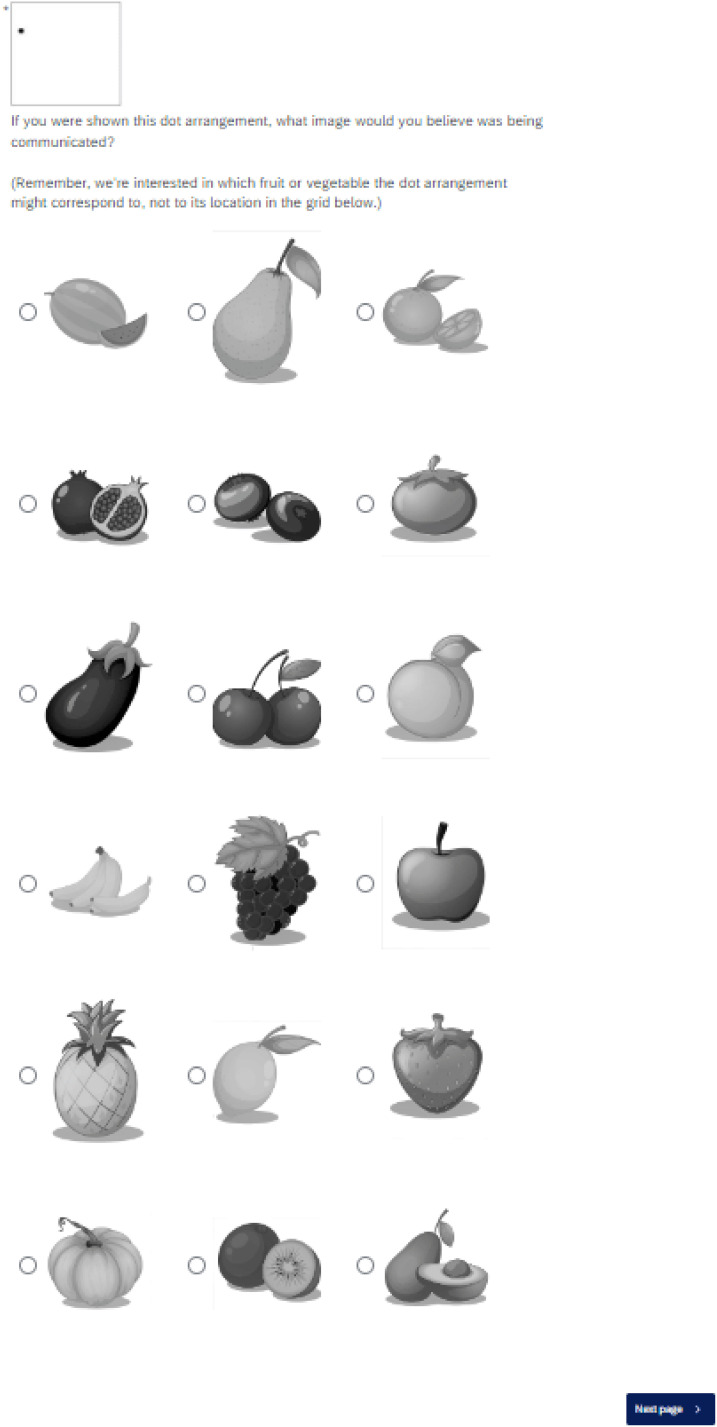
A sample question from Condition 4.

**Figure B5.**
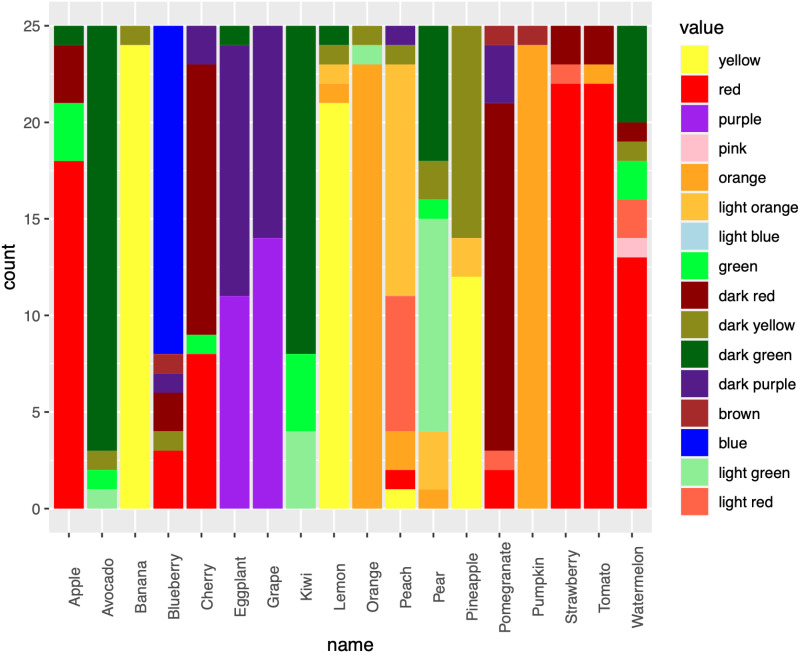
Results from Normalization Study, Condition 1.

**Figure B6.**
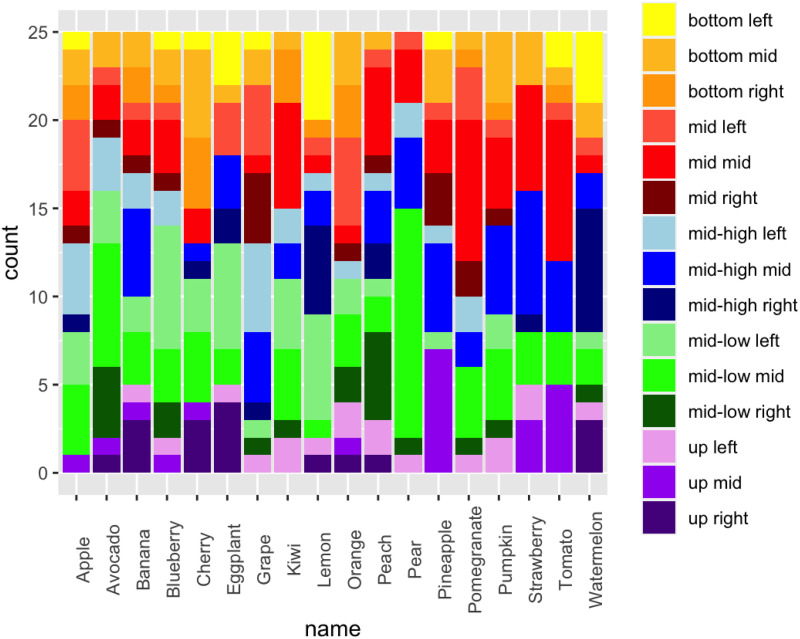
Results from Normalization Study, Condition 2.

**Figure B7.**
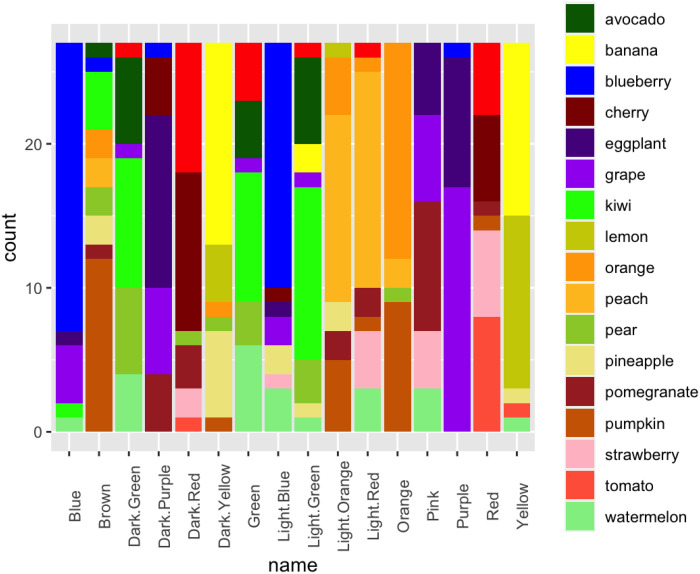
Results from Normalization Study, Condition 3.

**Figure B8.**
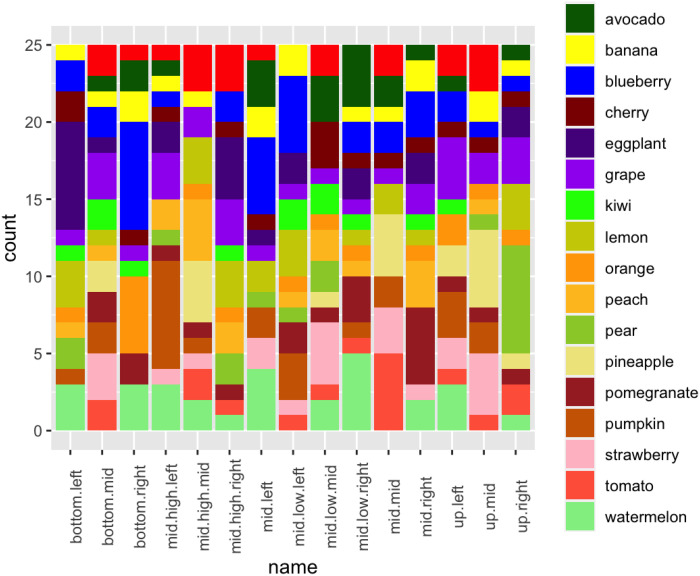
Results from Normalization Study, Condition 4.

**Figure D1.**
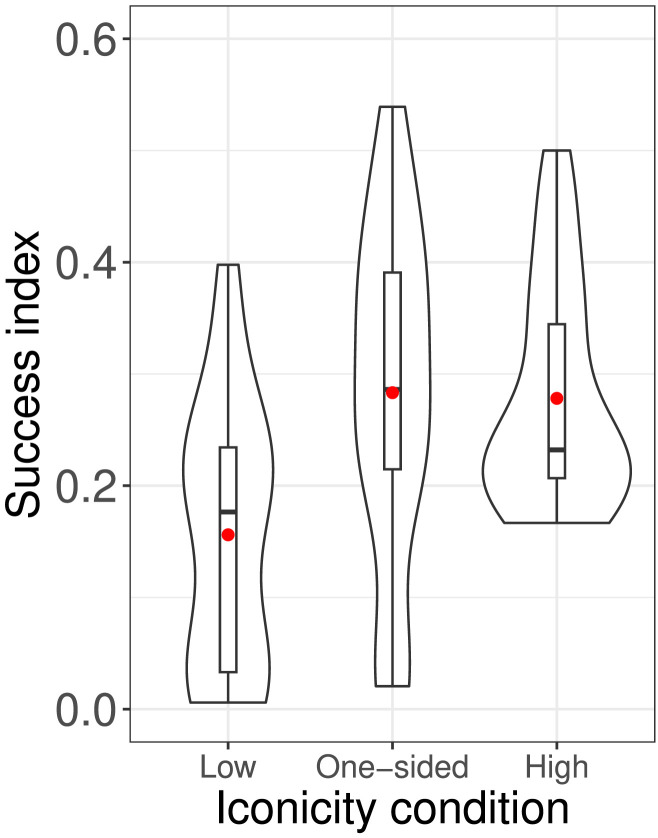
Success index with no practice rounds or dummy rounds.

**Figure D2.**
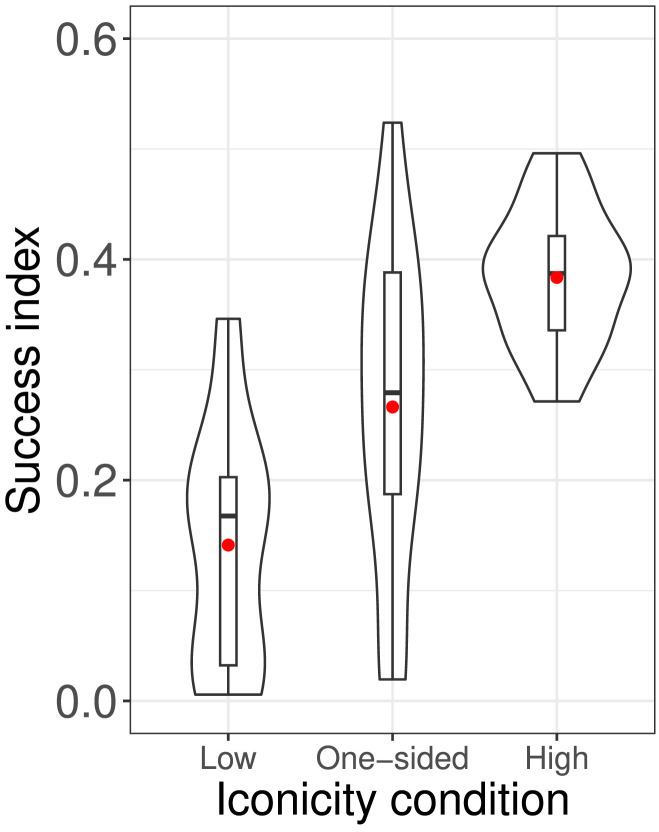
Success index with practice rounds but no dummy rounds.

**Figure E1.**
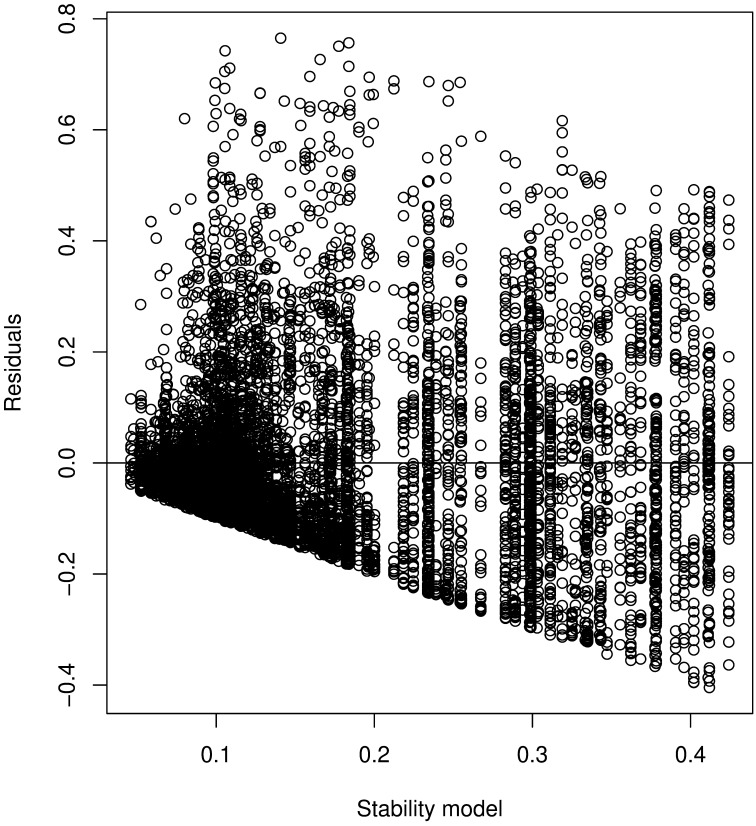
Residuals plot for stability model.

**Figure E2.**
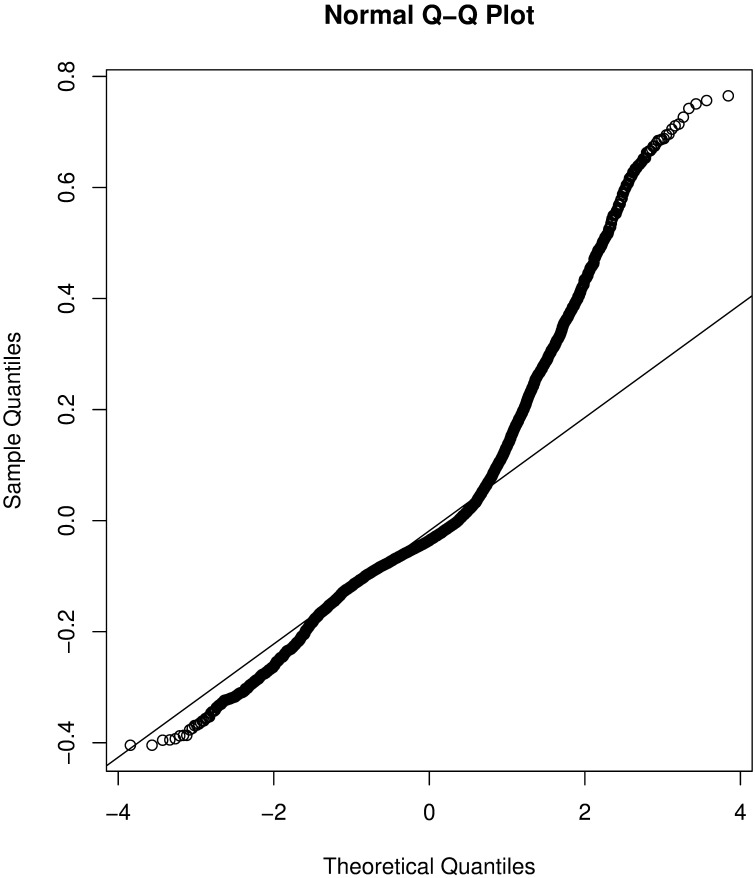
Q–Q plot for stability model.

**Figure E3.**
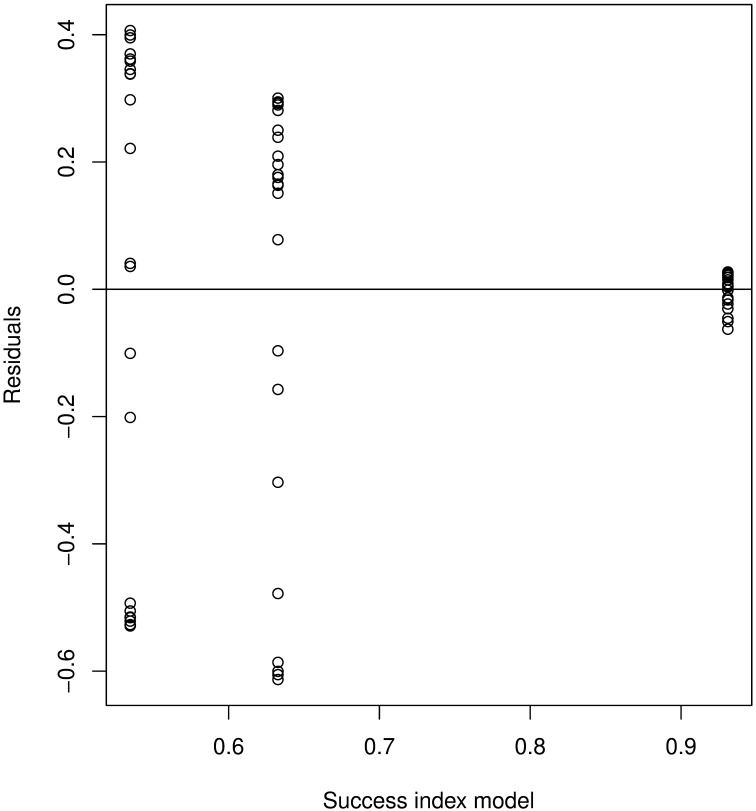
Residuals plot for success index (including practice and dummy rounds).

**Figure E4.**
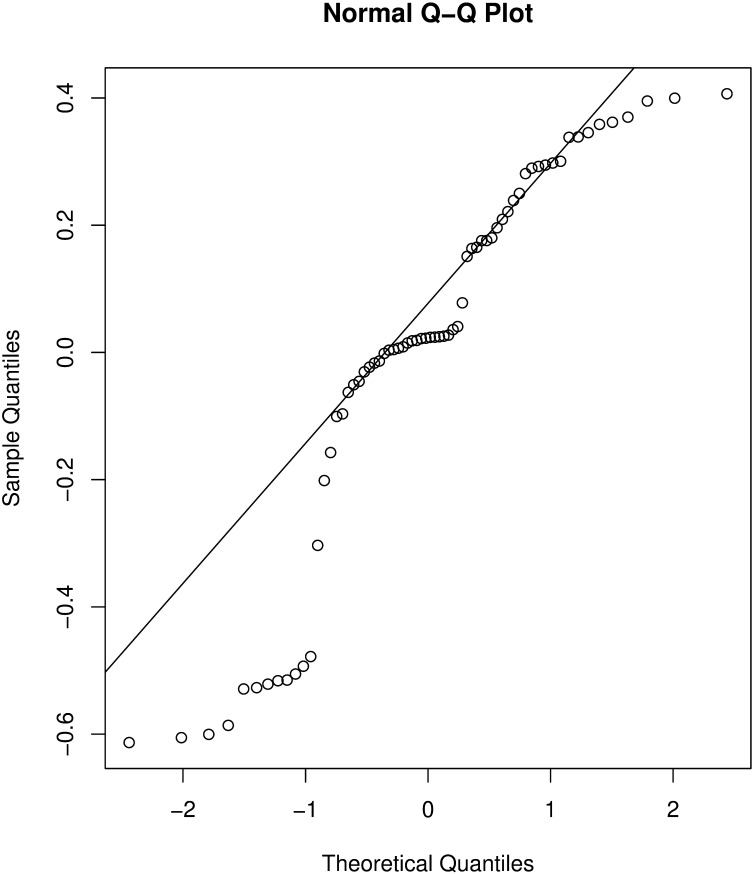
Q–Q plot for success index (including practice and dummy rounds).

**Figure E5.**
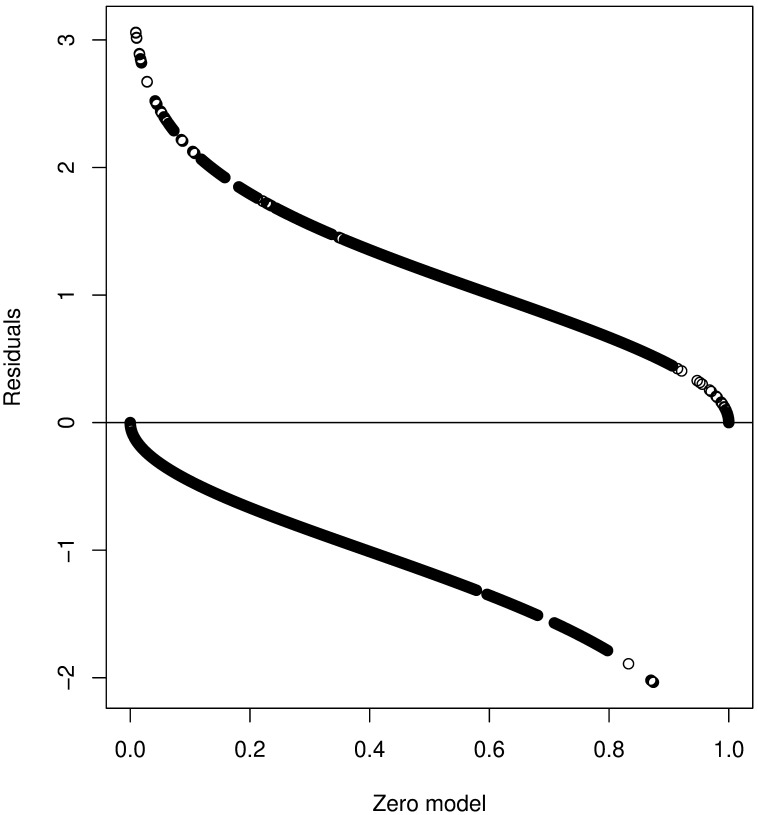
Residuals plot for referent presence model.

**Figure E6.**
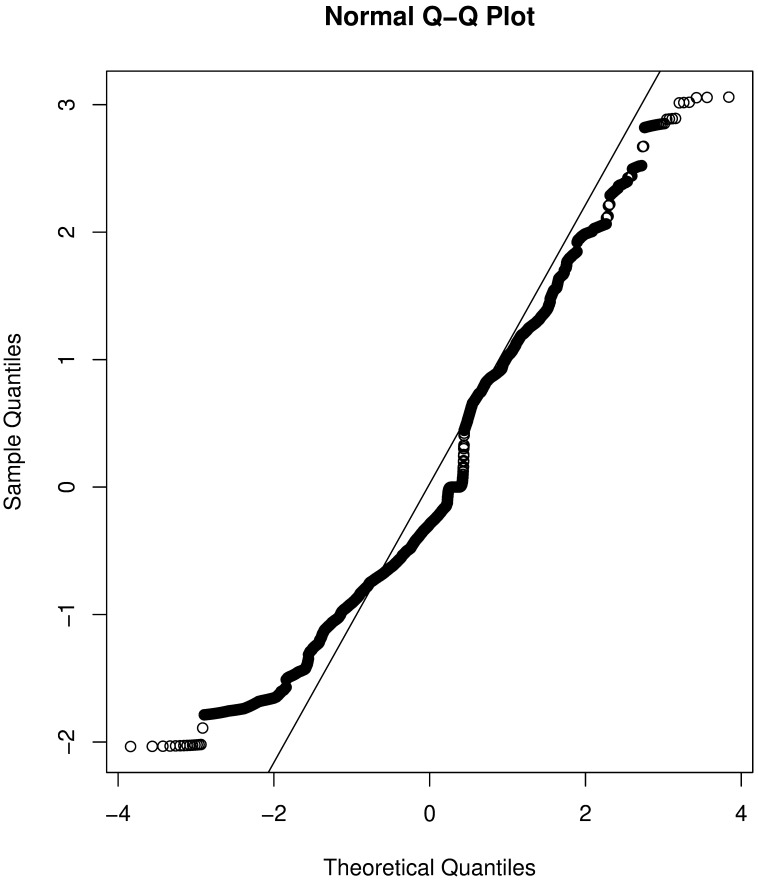
Q–Q plot for referent presence model.

**Figure E7.**
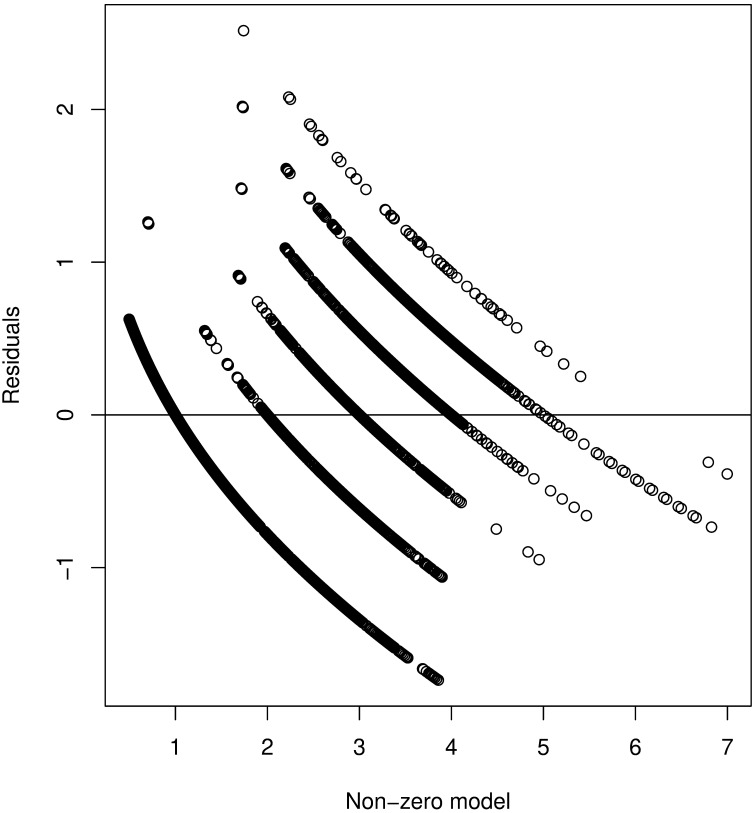
Residuals plot for non-zero referent model.

**Figure E8.**
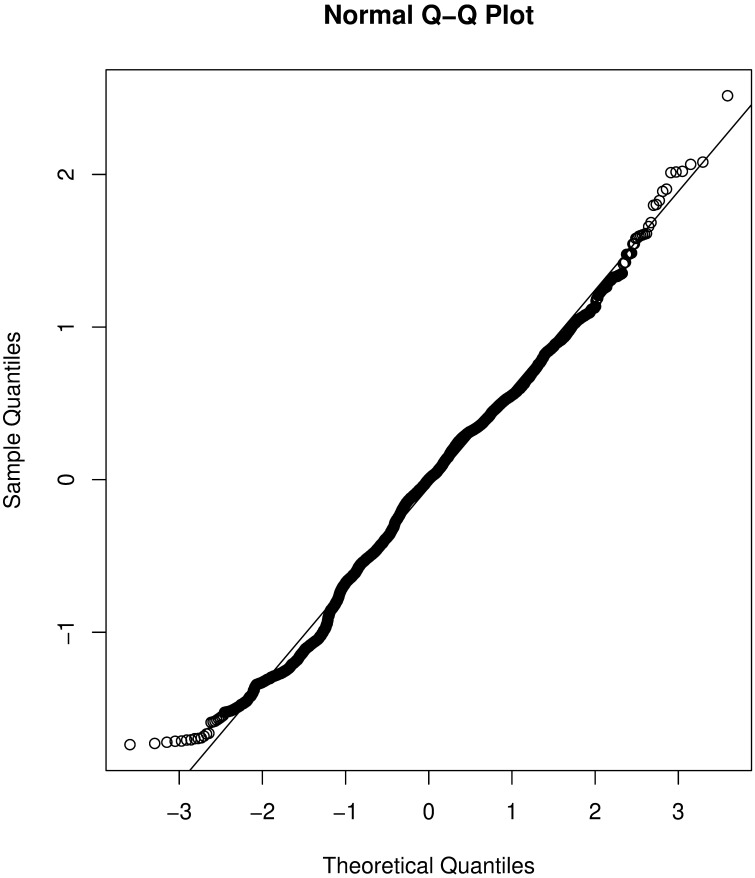
Q–Q plot for non-zero referent model.

## APPENDIX C: INSTRUCTIONS

Below we present instructions that were presented to all participants. Square brackets indicate text that varied depending on whether the participant in question would see dots or colors.

Sender Instructions:

Welcome! You’re going to be playing a cooperative guessing game with one partner.

You will see several fruits and vegetables in this section on the left of the screen. The one in the center will be highlighted. Your partner will see the same fruits and vegetables, but in **DIFFERENT POSITIONS**.

Your job is to try to convey to your partner which fruit or vegetable is highlighted.

Note that, on every round, you will be conveying a fruit or vegetable to your partner, and your partner will be guessing which it is—**these roles will never switch.**

You will attempt to convey the fruit or vegetable by selecting a single [DOT IN A PARTICULAR POSITION/COLOR] to send. You can do this by moving a finger on the trackpad. Your finger’s position will correspond to a [dot/color], which will be displayed in the top-right section of the screen. You can select one [dot position/color] to send to your partner. This works as follows. Please read carefully!

1. You can only have one finger on the trackpad at any one time or [dots/colors] will not show.
2. You can select the current [dot position/color] by HOLDING YOUR FINGER IN THE SAME LOCATION for 1 s (The small panel below will show the [dot position/color] to confirm that it has been selected).
3. When you have selected the [dot/color] you want, LIFT YOUR FINGER FROM THE PAD TO SEND IT to your partner as a message.

**Remember that your partner’s fruits and vegetables are NEVER in the same places as your fruits and vegetables! You will NEVER be able to convey the fruit or vegetable based on its position.**

When your partner has received the message you sent, they will attempt to select the correct fruit or vegetable. Then you’ll receive feedback: The fruit or vegetable that your partner chose will be highlighted. If they choose the right fruit or vegetable (i.e., the one that you have in the center) your joint score will increase.

The game might seem difficult at first! But you’ll get the hang of it with practice! To give you some extra practice, the first few turns will last a little longer.

The entire game will not take longer than an hour. If you do very well, it will END EARLY!

Please ask now if you have any questions.

When you are ready to start, let the experimenter know.

Receiver Instructions:

Welcome! You’re going to be playing a cooperative guessing game with one partner.

You will see several fruits and vegetables in this section on the left of the screen, with a cursor in the center. Your partner will see the same fruits and vegetables, but in **DIFFERENT POSITIONS**.

Your partner’s job for each round is to try to convey to you one of the displayed fruits or vegetables (which will be chosen for them).

Note that, on every round, your partner will be conveying a fruit or vegetable to you, and you will be guessing which it is—**these roles will never switch.**

They will attempt to convey the fruit of vegetable by sending you a SINGLE [DOT IN A PARTICULAR POSITION/COLOR]in the space on the top right. Use this [dot position/color] to try to guess what fruit or vegetable your partner is trying to convey to you. When you are ready to guess, use the arrow keys to move the cursor and choose the fruit or vegetable, then press return to select it (DO NOT FORGET TO PRESS RETURN)! You can only make your guess after you have received a [dot/color] from your partner.

**Remember that your partner’s fruits and vegetables are NEVER in the same places as your fruits and vegetables! You will NEVER be able to guess the fruit or vegetable based on the fruit or vegetable’s position on your screen.**

After you’ve guessed, you’ll receive feedback: The fruit or vegetable that your partner was trying to convey will be highlighted. If you choose the correct fruit or vegetable, your joint score will increase.

The game might seem difficult at first! But you’ll get the hang of it with practice! To give you some extra practice, the first few turns will last a little longer.

The entire game will not take longer than an hour. If you do very well, it will END EARLY!

Please ask now if you have any questions.

When you are ready to start, let the experimenter know.

Sender Reminder Instructions:

Practice rounds over!

Subsequent rounds will last 30 seconds each.

Remember:

1. Your partner’s fruits and vegetables are NEVER in the same places as yours. You will NEVER be able to convey a fruit or vegetable based on its position.
2. If you do very well, the game will END EARLY!

Please ask now if you have any questions.

Receiver Reminder Instructions:

Practice rounds over!

Subsequent rounds will last 30 seconds each.

Remember:

1. Your partner’s fruits and vegetables are NEVER in the same places as yours. You will NEVER be able to guess a fruit or vegetable based on position.
2. If you do very well, the game will END EARLY!

Please ask now if you have any questions.

## APPENDIX D: COMPARISON OF SUCCESS INDEX RESULTS

The figures below illustrate how the success index is influenced by the inclusion or exclusion of practice and dummy rounds. In Figure D1, results are shown with neither practice nor dummy rounds included. This severely underestimates success in the High iconicity condition. It also benefits dyads in the One-sided iconicity condition relative to those in the Low-iconicity condition.

In Figure D2, practice rounds (but no dummy rounds) are included. A comparison with Figure D1 illustrates how much was already achieved during the practice rounds for dyads in the High-iconicity condition. Nevertheless, without dummy rounds, the success of dyads in the High iconicity condition is still underestimated, as demonstrated by a comparison between Figure D2 on one hand and Figure 7, or even non-success index measures like in Figure 6b, on the other.
